# Characterization of endophytic bacteriome diversity and associated beneficial bacteria inhabiting a macrophyte *Eichhornia crassipes*

**DOI:** 10.3389/fpls.2023.1176648

**Published:** 2023-06-19

**Authors:** Di Fan, Timothy Schwinghamer, Shuaitong Liu, Ouyuan Xia, Chunmei Ge, Qun Chen, Donald L. Smith

**Affiliations:** ^1^ School of Biology, Food and Environment, Hefei University, Hefei, China; ^2^ Lethbridge Research and Development Centre, Agriculture and Agri-Food Canada, Lethbridge, AB, Canada; ^3^ Department of Plant Science, McGill University, Sainte-Anne-de-Bellevue, QC, Canada

**Keywords:** endophytes, bacterial community, diversity analysis, *Eichhornia crassipes*, beneficial bacteria

## Abstract

**Introduction:**

The endosphere of a plant is an interface containing a thriving community of endobacteria that can affect plant growth and potential for bioremediation. *Eichhornia crassipes* is an aquatic macrophyte, adapted to estuarine and freshwater ecosystems, which harbors a diverse bacterial community. Despite this, we currently lack a predictive understanding of how *E. crassipes* taxonomically structure the endobacterial community assemblies across distinct habitats (root, stem, and leaf).

**Methods:**

In the present study, we assessed the endophytic bacteriome from different compartments using 16S rRNA gene sequencing analysis and verified the *in vitro* plant beneficial potential of isolated bacterial endophytes of *E. crassipes*.

**Results and discussion:**

Plant compartments displayed a significant impact on the endobacterial community structures. Stem and leaf tissues were more selective, and the community exhibited a lower richness and diversity than root tissue. The taxonomic analysis of operational taxonomic units (OTUs) showed that the major phyla belonged to Proteobacteria and Actinobacteriota (> 80% in total). The most abundant genera in the sampled endosphere was *Delftia* in both stem and leaf samples. Members of the family Rhizobiaceae, such as in both stem and leaf samples. Members of the family Rhizobiaceae, such as *Allorhizobium- Neorhizobium-Pararhizobium-Rhizobium* were mainly associated with leaf tissue, whereas the genera *Nannocystis* and *Nitrospira* from the families Nannocystaceae and Nitrospiraceae, respectively, were statistically significantly associated with root tissue. *Piscinibacter and Steroidobacter* were putative keystone taxa of stem tissue. Most of the endophytic bacteria isolated from *E. crassipes* showed *in vitro* plant beneficial effects known to stimulate plant growth and induce plant resistance to stresses. This study provides new insights into the distribution and interaction of endobacteria across different compartments of *E. crassipes* Future study of endobacterial communities, using both culture-dependent and -independent techniques, will explore the mechanisms underlying the wide-spread adaptability of *E. crassipes*to various ecosystems and contribute to the development of efficient bacterial consortia for bioremediation and plant growth promotion.

## Introduction

Plants interact with a multitude of microorganisms that colonize the phytosphere (Yang et al., 2013), where mutually beneficial unions may form. Empirical studies have suggested that, bacteria, one of the dominant components of plant microbiota, can affect plant growth and stress responses (Abadi et al., 2020; Fan and Smith, 2021). Harnessing these biological processes can provide a strategy for increasing plant fitness and productivity, ultimately leading to climate resilience for both agriculture and the environment (Tian et al., 2020; Pham et al., 2022).

Endophytic bacteria are those residing in internal plant tissues and many of them exert no negative effects on the host plant performance. The beneficial effects of endophytes on plant growth and health were investigated extensively. Plant growth promoting (PGP) endophytic bacteria enhance plant growth via nitrogen fixation (Yang et al., 2020), stress alleviation (Fan and Smith, 2021), the production of phytohormones (Duca et al., 2014) and production of growth-promoting signal compounds (Prudent et al., 2015). Bacterial endophytes can induce pollutant tolerance, therefore they were candidates for utilization in bioremediation (Pham et al., 2022). Besides terrestrial plants, there have been many studies indicated that culturable endobacteria in wetland plants can be used for bioremediation, enzyme production (Ho et al., 2012), biocontrol (Shcherbakov et al., 2013), and plant growth promotion (Zhang et al., 2014a). For example, endobacteria (i.e. species of *Delftia*, *Staphylococcus*, and *Aeromonas*) that were isolated from three aquatic plants, *Potamogeton crispus*, *Nymphaea tetragona*, and *Najas marina* degraded insecticides, such as chlorpyrifos and fenpropathrin (Chen et al., 2012). Endobacterial strains (*Pseudomonas* and *Bacillus* sp.) isolated from the root and stem of *Scirpus triqueter* can degrade diesel fuel, and they are candidates for the remediation of oil-contaminated environments (Zhang et al., 2014b). An endophytic bacterium, *Enterobacter* sp. SVUB4, isolated from roots of *Eichhornia crassipes*, accumulated heavy metals and promoted plant growth (El-Deeb et al., 2012).

Distinct bacteriomes were isolated and found in association with terrestrial and aquatic plant species (Chen et al., 2012; Zhang et al., 2015; Fan et al., 2018), which indicated that bacteria are filtered from those present in the environment surrounding the plant, and then supported by host plants. The fact that only a minority of bacteria can be isolated and cultivated in the laboratory limited our attempt to quantify and characterize bacterial diversity and the interactions between plants and bacteriomes. Second generation sequencing techniques enabled large-scale analysis of abundance, taxonomic structure, and potential functional features of plant-associated bacterial communities (Khare et al., 2018; Lucaciu et al., 2019). There has been some research studying the evolution, adaptation, and interaction of bacteria associated with host plants. The effects of plant niches (fruit, leaf, stem, and root) on endobacterial community structures were studied in *Macleaya cordata* (Lei et al., 2021). Another report studied dissimilarities between the rhizosphere, endosphere, and phyllosphere of two Antarctic vascular plants, in regard to the diversity, taxonomic assignments, and predicted function of plant-associated bacteria (Zhang et al., 2020). Differences in bacterial diversity and composition between water, soil, and root endospheres of *Typha orientalis* were observed by Wang et al. (2023) and, during plant growth, more PGP bacteria were observed in the root endosphere than in rhizosphere soil. The effect of oil sands on the rhizosphere and endosphere bacterial assembly in *Hordeum vulgare* and *Meliotus albus* was also analyzed, showing that the root endobacteriome was a subset of the rhizosphere community and the selection process was driven by plant factors (Mitter et al., 2017).

Water hyacinth (*Eichhornia crassipes*) is an aquatic floating weed present throughout the tropical and subtropical world, with a high reproductive capacity. *E. crassipes* can be used for animal feed, biofuel production (Ayanda et al., 2020), and pharmaceuticals (Khalid et al., 2020), and it affects the ecological characteristics of freshwater ecosystems through attributes such as phytoremediation potential (Gaballah et al., 2021; Singh and Balomajumder, 2021). Previous culture-dependent studies revealed that *E. crassipes* harbored a wide diversity of rhizosphere (Zheng and Fu, 2015; Lafta Alzurfi and Katia, 2021), endosphere (Lan et al., 2008), phyllo- and root-endosphere bacteria (Shehzadi et al., 2016). Lan et al. (2008) collected 56 endobacterial strains from the whole plant of *E. crassipes*, using 20 different culture media. These endophytes were classified into 32 genera, and *Microbacterium* was the most abundant genus (Lan et al., 2008). When inoculated with microbes, the arsenic uptake potential by *E. crassipes* was enhanced (Kaur et al., 2018). Although there were some reports of culture-independent bacterial diversity of aquatic plants, such as *Phragmites australis* (Li et al., 2010), *Typha angustifolia* (Li et al., 2011), and *Typha orientalis* (Wang et al., 2023), most previous studies on plant-associated bacteriomes focused on the terrestrial ecosystem, and there remain gaps in our knowledge concerning bacterial colonization of plants that are associated with water-based systems. The bacterial assembly and interactions with *E. crassipes* (Fu and Zheng, 2016; Duraivadivel et al., 2020; Sharma et al., 2021) are for the most part unknown (rhizosphere and rhizoplane). As such, the present study was undertaken to: (1) systematically investigate the endobacterial community diversity and structures associated with root, stem, and leaf of *E. crassipes*, based on both culture-dependent method and high-throughput sequencing techniques; (2) identify the keystone taxa of *E. crassipes*-associated bacteriomes; and (3) assess potential plant and environment beneficial activities of isolated bacterial strains from *E. crassipes*. Functional traits of the endobacteriome were also predicted.

## Materials and methods

### Study site and sample collection

To investigate the spatial distributions of endophytic bacterial communities in *E. crassipes*, samples were collected in October 2021 in Hefei (30°56′-32°33′ N, 116°40′-117°58′E), Anhui province, China. Hefei has an average elevation of 37.51 m above sea level and is characterized by a temperate climate with an annual precipitation of 1000 mm distributed mainly from May to August and an average annual temperature of 15.7°C. July is the hottest month with an average temperature of 32.0°C, while January is the coldest month (6.8°C).

*E. crassipes* were checked for any physical impurities and selected based on vigorous growth habit. A total of 24 intact plants were individually collected, wrapped in plastic bags, and immediately transported to the laboratory. Six plants were pooled for each replicate. Lake water was sampled concomitantly, in which 5-L water (one replicate) was collected in sterile plastic bottles and transported to the laboratory on ice. The samples were processed immediately.

### Sample preparation

Lake water was filtered through 0.22-μm polycarbonate membranes, to capture microbes which were stored at -80°C until DNA extraction. To collect the endophytic bacteria, leaf/stem/root samples from each of the replicates were thoroughly washed with tap water and then rinsed in ddH_2_O until the water was clear. A further cleaning was conducted using 10% Sparkleen™ 1 (Fisherbrand™) water solution followed by several rinses in ddH_2_O (Fan et al., 2018). Plant samples were surface sterilized consecutively by immersion for 5 min in 70% ethanol, three rinses in sterile ddH_2_O, and 3 min shaking in 1% sodium hypochlorite, followed by six changes of sterile ddH_2_O. Aliquots of ddH_2_O from the last wash were plated on King’s B (KB), R2A, Tryptone Soya Broth (TSB), Lysogeny broth (LB), and 0.1 × LB agar plates, incubated at 28°C for 3 to 7 d, and then checked for microbial growth for the confirmation of surface disinfection.

### Water quality testing

The physicochemical parameters of water were determined by Guoke Testing Technology Co. Ltd (Hefei, China). Parameters including pH, total phosphorus (TP), ammonia nitrogen (NH_3_-N), sulfates (SO_4_^2-^), nitrates (NO^3-^), nitrite (NO^2-^), potassium (K), iron (Fe), sodium (Na), cadmium (Cd), lead (Pb), nickel (Ni), calcium (Ca), magnesium (Mg), chromium (Cr), arsenic (As), COD, BOD_5_ and TOC were evaluated to determine lake water quality (**Table S1**), which met the Class I of the National Surface Water Quality Standards (GB3838-2002).

### Isolation of cultivable endophytic bacteria

Population densities of endophytic bacteria in the plant samples were determined using conventional culture methods (Fan et al., 2018). Plant samples (leaf/stem/root) treated as above were chopped and macerated in sterile phosphate-buffered saline (PBS). Then the supernatants were empirically diluted in 10-fold series in PBS and the subsequent diluted sample (100 μL) was distributed in triplicate onto agar medium and incubated at 28°C for 2-5 days, after which colony forming units (CFU) g^-1^ were observed. Morphologically different colonies were selected based on growth rate, size, margin, shape and color etc. and purified three times on the original medium. Individual colonies were sub-cultured and frozen in the appropriate growth medium containing 25% glycerol at -80°C pending further processing.

### Taxonomic identification of isolated endophytic bacteria

DNA was extracted from isolates using a Bacterial DNA Kit (Omega Bio-Tek) following the protocol of the manufacturer. PCR of 16S rRNA genes and sequencing were conducted by Majorbio Company (Shanghai, China). In brief, the almost complete bacterial 16S rRNA gene was amplified with Ex Taq^®^ (Takara Bio Inc.), using primers 27F (5’-AGAGTTYGATCCTGGCTCAG-3’) and 1492R (5’- GGTTACCTTGTTACGACTT-3’). Conditions comprised an initial denaturation at 95°C for 5 min, followed by 25 cycles of 95°C for 30 s, 56°C for 30 s, 72°C for 1.5 min, and a final extension step at 72°C for 10 min on the last cycle. The amplicons were purified and sequenced on a 3730XL DNA analyzer system (Applied Biosystems). Retrieved DNA sequences were manually edited using Seqman Pro (DNASTAR Lasergene package). The similarities of 16S rRNA genes between isolated endophytes and their phylogenetic neighbors were determined through the EzBioCloud Database (access time: January 2023) (Yoon et al., 2017), supplemented with the BLASTN (https://blast.ncbi.nlm.nih.gov/Blast.cgi) of the NCBI to search for the closest matching sequences.

### Screening of endophytic bacterial isolates for PGP traits

The 55 isolated bacterial strains were assessed for general PGP traits. Activities of protease, cellulase (Weinert et al., 2010) and chitinase (Berg et al., 2001) was determined. Furthermore, the isolated strains were tested for mineral phosphate solubilization (Nautiyal, 1999), organic phosphate solubilization (Li et al., 2019), 1-aminocyclopropane-1-carboxylate (ACC) deaminase activity (Penrose and Glick, 2003) and production of indole-3-acetic acid (IAA) (Gordon and Weber, 1951) and siderophore (Alexander and Zuberer, 1991). All isolates were also tested for heavy metal (As(III)) resistance (Sun et al., 2020) and plastic degrading ability (Shah et al., 2013). All the experiments were repeated three times, with four replicates each time.

### DNA extraction, PCR amplification, and amplicon sequencing

Genomic DNA was extracted from plant and water samples prepared as described above. DNA quality and quantity were estimated by NanoDrop2000 (NanoDrop Technologies, Inc.). The V5-V7 region of the endophytic bacterial 16S rRNA gene was amplified by two rounds of nested PCR in ABI GeneAmp^®^ 9700. The first amplification was conducted using primers 799F (5′-AACMGGATTAGATACCCKG-3′) and 1392R (5′-ACGGGCGGTG TGTRC-3′) under the following conditions: 95°C for 3 min, 27 cycles (95°C for 30 s, 55°C for 30 s and 72°C for 45 s). The conditions for the second amplification were 95°C for 3 min, 13 cycles (95°C for 30 s, 55°C for 30 s and 72°C for 45 s), using primers 799F and 1193R (5′-ACGTCATCCCCACCTTCC-3′). PCR amplification of the V3-V4 region of bacterial 16S rRNA genes from water samples was conducted using primers 338F (5′- ACTCCTACGGGAGGCAGCAG-3′) and 806R (5′-GGACTACHVGGGTWTCTAAT-3′). PCR reactions were performed in 20-μL mixtures containing 4 μL of 5 × FastPfu Buffer, 2 μL of 2.5 mM dNTPs, 0.8 μL of each primer (5 μM), 0.4 μL of TransStart FastPfu DNA polymerase, 0.2 μL of BSA, 10 ng of DNA template, and nuclease-free water. The corresponding products were checked on 2% agarose gels. The purified amplicons were paired-end sequenced on an Illumina MiSeq platform at Majorbio Company with the strategies of PE250 (paired-end 250 bp). The sequencing data was submitted to the NCBI Sequence Read Archive (SRA) under the accession number PRJNA947841.

### Sequence processing

The paired-end reads were merged using fast length adjustment of short reads (FLASH) software (Magoč and Salzberg, 2011), after which the 16S rRNA gene sequences were processed and filtered by the Quantitative Insights Into Microbial Ecology (QIIME) pipeline (Caporaso et al., 2010) to get high quality sequences. Chimera was filtered using the UPARSE pipeline (Edgar, 2013). The above-preprocessed sequences with ≥ 97% similarity were clustered into the same operational taxonomic units (OTUs), with a representative sequence generated for each OTU. The Ribosomal Database Project (RDP) Classifier algorithm assigned each representative OTU to taxonomic ranks against SILVA Database (confidence threshold: 0.7). For statistical analyses of bacterial communities in plant and water samples, sequences of each sample were normalized to 68,176 and 66,479 sequences, respectively, based on the sample with the lowest sequences. To remove the effect of non-microbiota, chloroplast and mitochondrial sequences were removed out of the OTU table in all samples.

### Bioinformatic and statistical analyses

Rarefaction analysis was conducted to compare the richness of OTUs and to evaluate the sequencing depth of each sample. The comparison of bacterial richness and diversity among samples were assessed using: alpha indexes, including Chao 1, Shannon, Simpson, Sobs, phylogenetic diversity (PD), abundance-based coverage estimate (ACE), and sample coverage, using the mothur program (Schloss et al., 2009). Principal coordinate analysis (PCoA) using unweighted UniFrac distance metrics for assessing the beta-diversity differences was carried out using the online tool of Majorbio Cloud Platform (https://cloud.majorbio.com/page/tools/), to quantify the dissimilarity of plant samples. In addition, partial least squares discriminant Analysis (PLS-DA), analysis of similarity (ANOSIM) and permutational multivariate analysis of variance (PERMANOVA) were also performed to identify whether plant compartmentation had significant effects on bacterial composition (Warton et al., 2012). To identify distinguishing taxa among groups, multi-level analysis was conducted by the linear discriminant analysis (LDA) effect size (LEfSe) (http://huttenhower.sph.harvard.edu/galaxy/root?tool_id=lefse_upload) and the results were visualized with cladograms and bar charts. *Post-hoc* comparisons were conducted by pairwise Wilcoxon rank-sum tests to further determine the pairwise differences. The Networkx package and Cytoscape were used to visualize network structures in the bacterial communities across samples. Organism level bacteriome phenotypes were predicted using BugBase pipeline (https://bugbase.cs.umn.edu) under the default parameters. The ecologically relevant functions of the bacteriome in different niches (water and endospheres of root, stem, and leaf) were predicted using FAPROTAX (Functional Annotation of Prokaryotic Taxa) analysis (www.loucalab.com/archive/FAPROTAX) based on the 16S rRNA gene sequencing data.

## Results

### General characteristics of sequencing data

After quality filtration and chimera checking of the endophytic bacterial microbiota sequences, 12 plant samples yielded 16S rRNA gene sequences with an average depth of sequencing of 90,858 reads per sample, ranging from 70,214 to 120,914 reads per sample with an average length of 375 bp. After normalization, a total of 818,112 sequences was obtained. In total, 2,426 OTUs of endophytic bacteria at a 97% sequence similarity level were identified, from which 32 phyla, 747 genera, and 1,292 species were detected. While for water samples, a core set of 265,916 sequences and 701 OTUs (at 3% evolutionary distance) was obtained. The bacteria were from 31 phyla, 361 genera and 516 species for the water bacteriome.

**Figure 1:**
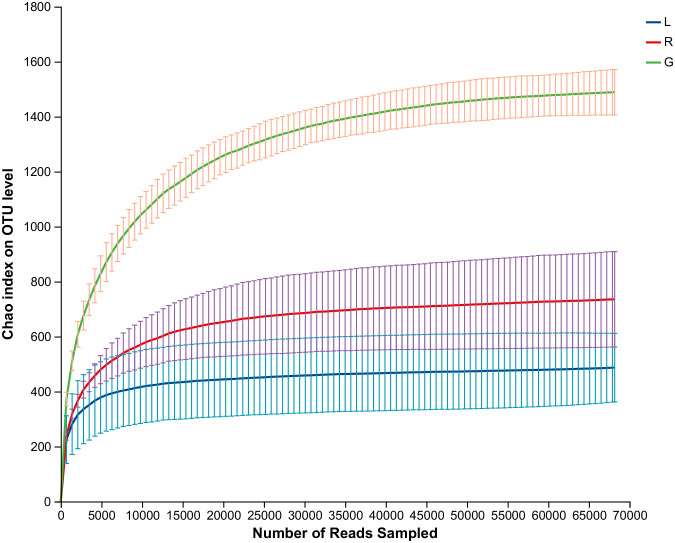
Rarefaction curves based on the 16S rRNA gene sequences of each sample at cutoff level of 3%. The error bars represent the standard error of four replicates. L, leaf endosphere; G, root endosphere; R, stem endosphere.

As shown in the rarefaction curves for the Chao1 biodiversity index (**Figure 1**), the number of OTUs increased sharply but gradually reached a plateau, indicating that the sequencing depth was sufficient and represented the structural characteristics of the bacteria well for plant samples, as the curves tended toward saturation. All samples had Good’s coverage values over 0.99, indicating that the sequencing results could well represent the bacterial communities in the plant endosphere (**Table 1**). As revealed by the Chao1 and Shannon index, the richness and diversity of the bacterial community in the root endosphere was significantly higher than in the stem and leaf endospheres. Furthermore, a comparison of other alpha diversity indexes showed a significant disparity in leaf samples compared with root samples (**Table 1**). For example, the richness indexes (Sobs and ACE) in root samples were 2.8- and 3.2-fold higher than leaf samples, respectively. The PD diversity estimator ranged from 88.3 to 97.3 in the root bacterial microbiome, and from 32.5 to 54.9 in the leaf bacteriome, indicating an average 2.2-fold increase in the root compared to leaf tissue. The Shannon index in root samples showed a 1.4-fold increase, compared to the leaf endosphere. There was no statistically significant difference, with respect to the six alpha diversity indices, between leaf and stem samples. Overall, there was a significant downward trend of the diversity of endophytic bacterial species in the root over leaf tissues.

**Table 1:** Summary of endobacterial alpha-diversity indexes of the samples from three plant niches (root-G, stem-R, and leaf-L).

Sample ID	Coverage (%)	Chao1	Shannon	Simpson	PD	Sobs	ACE
L01	99.974	323.250	2.317	0.410	32.463	285	296.322
L02	99.968	623.044	4.150	0.050	54.948	613	619.395
L03	99.884	481.018	3.649	0.047	38.225	425	476.599
L04	99.956	520.917	3.593	0.120	46.023	504	516.595
**Average**	99.946a	487.057b	3.427b	0.157a	42.915b	456.75b	477.228b
R01	99.746	947.664	4.207	0.034	66.189	816	946.454
R02	99.861	749.197	3.918	0.056	58.340	676	730.274
R03	99.959	523.539	3.298	0.186	46.562	509	519.454
R04	99.852	721.158	3.912	0.056	58.684	668	728.439
**Average**	99.854a	735.389b	3.833b	0.083ab	57.444b	667.25b	731.155b
G01	99.545	1570.615	5.063	0.017	96.498	1325	1581.737
G02	99.619	1472.031	5.011	0.018	97.333	1325	1512.691
G03	99.579	1380.755	4.500	0.042	88.270	1167	1425.454
G04	99.539	1531.483	4.846	0.024	94.777	1287	1568.677
**Average**	99.571b	1488.721a	4.855a	0.025b	94.220a	1276a	1522.140a

The statistical significance was assessed by Wilcoxon rank-sum test (P < 0.05). Different lowercase letters showed significant differences.

### Analyses of bacterial community composition and taxonomy at different levels

**Figure 2:**
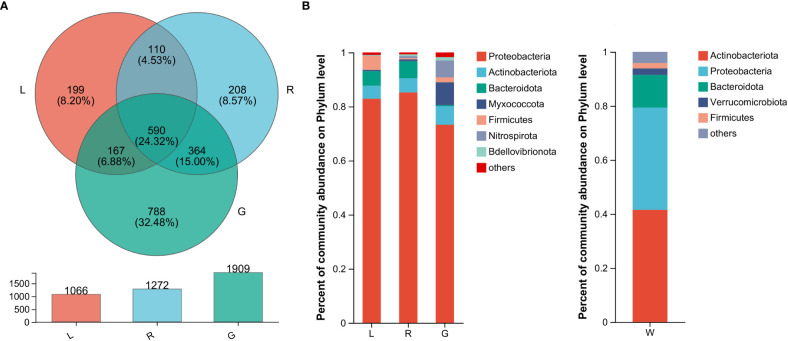
The bacterial microbiota profiles of samples from *Eichhornia crassipes*. **(A)** Venn diagram illustrating the overlap of the OTU calculated separately for the bacterial communities in different plant niches. The relative abundance of dominant phyla **(B)** in the leaf, stem, root, and water groups (G, root endosphere; R, stem endosphere; L, leaf endosphere; W, water).

**Figure 3:**
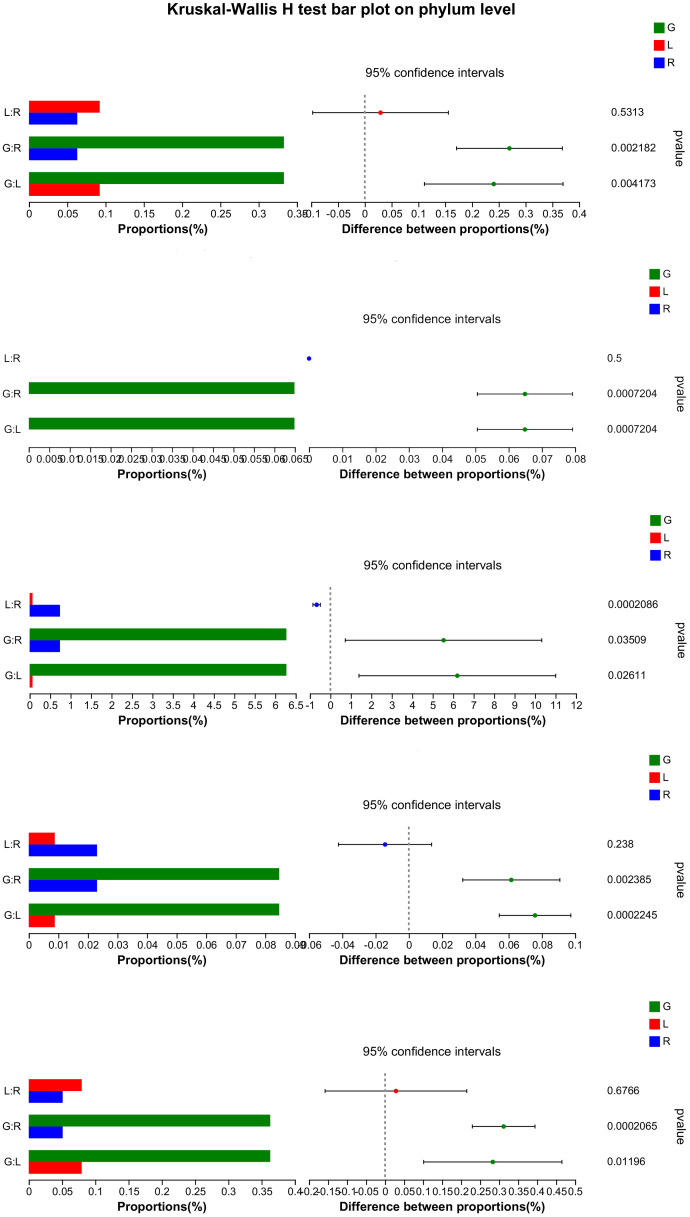
Significance test (Kruskal-Wallis) for differences in bacterial phylum among endosphere of root, stem, and leaf of *E. crassipes*. The X-axis on the left side indicates the mean relative abundance in different groups of species.

In order to verify our hypothesis that host plant compartmentation can influence the composition of the endobacteriome, taxonomic analyses were conducted. The Venn diagram revealed that OUTs differed among the three plant niches (**Figure 2A**). There were 590 shared OTUs, defined as generalists, among the three groups, with L-R, L-G, and R-G each having 110, 167, and 364 unique OTUs, respectively. There were 788, 208, and 199 OTUs exclusive to G, R, and L samples. The top 7 phyla (> 1% of relative abundance in each sample) are shown in **Figure 2B**. Generalist OTUs consisted of 22 phyla, including Proteobacteria, Actinobacteriota, Bacteroidota, Myxococcota, Firmicutes, and Nitrospirota as the top six, making up for more than 90% of all bacteria in each group; whilst in water samples, Actinobacteriota, Proteobacteria, Bacteroidota, Verrucomicrobiota, and Firmicutes were among the top 5 phyla (**Figure 2B**). Myxococcota (8.43%) and Nitrospirota (6.27%) were more abundant in the below-water surface compartment, roots, while Bacteroidota and Verrucomicrobiota were widely distributed in the above-water surface (stem and leaf; 5.37% - 6.26%) and water (2.45%), respectively. Actinobacteriota were more numerous (41.54%) in the water bacteriome as compared to plant endobacteriome (< 6.85%). At the phylum level, Verrucomicrobiota, Planctomycetota, Dependentiae, and Chloroflexi were significantly decreased in stem and leaf niches, while Firmicutes exhibited significantly higher abundances in leaf endosphere than in other plant niches. Bacteroidota was significantly enriched in stem (stem vs. root). Nitrospirota showed a tendency toward gradual change from root, stem to leaf (*P* < 0.05) (**Figure 3**).

**Figure 4:**
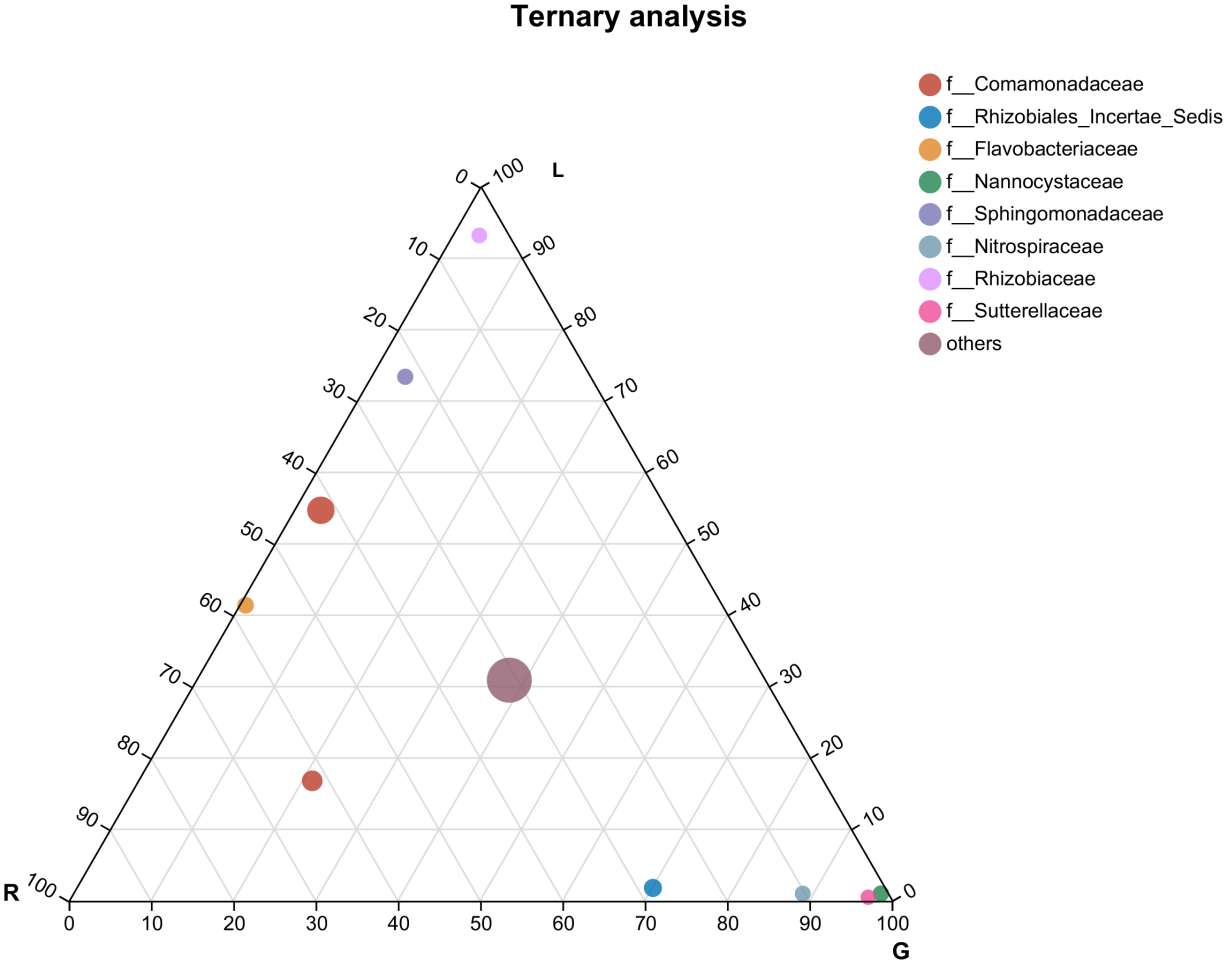
Ternary plot representing the relative occurrence of individual genus (circles) in leaf, stem, and root samples of *E. crassipes* that are presented at the family level. L, leaf; R, stem; G, root.

According to the most abundant families (**Figure 4**), the leaf endosphere harbored a high abundance of Rhizobiaceae and Sphingomonadaceae, whereas in root endosphere, Nannocystaceae, Nitrospiraceae, and Sutterellaceae were the dominant families. Genera from the family Comamonadaceae were mostly associated with all bacterial endophytic communities, as well as water samples (**Figures 4**, **S1**), whereas Flavobacteriaceae was associated with the endosphere bacterial communities in both stem and leaf. Rhozobiales Incertae Sedis was mostly associated with stem and root. The most numerous three classified families in water were Sporichthyaceae (28.6%), Moraxellaceae (15.2%), and Ilumatobacteraceae (9.2%) (**Figure S1**).

**Figure 5:**
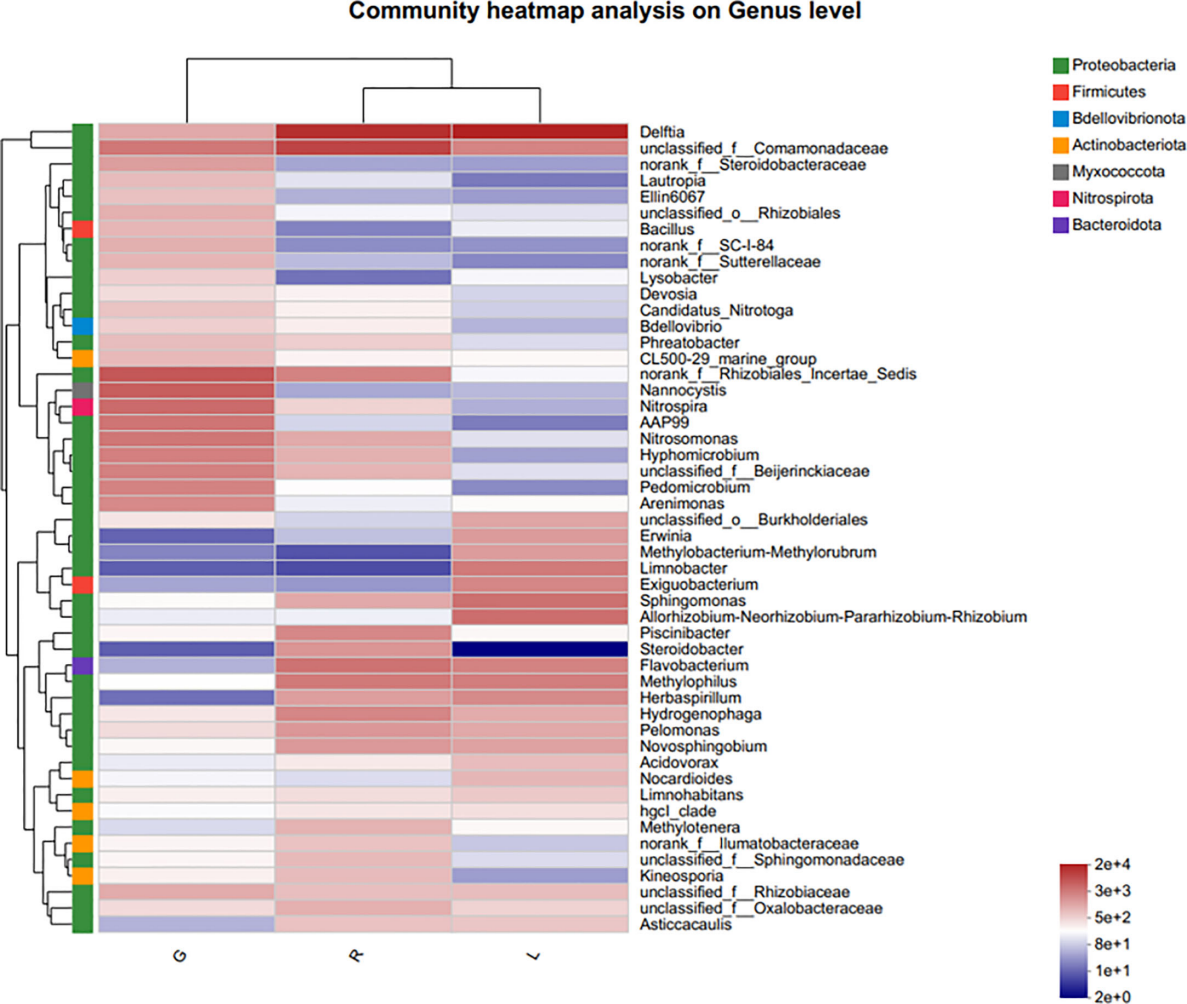
Indicator heatmap showing taxon-treatment-association strength of top 50 bacterial genera associated with different niches (endosphere) of *E. crassipes*. G, L, and R refer to the root, leaf, and stem samples, respectively. The value of each bar represents the mean of *n* = 4.

**Figure 6:**
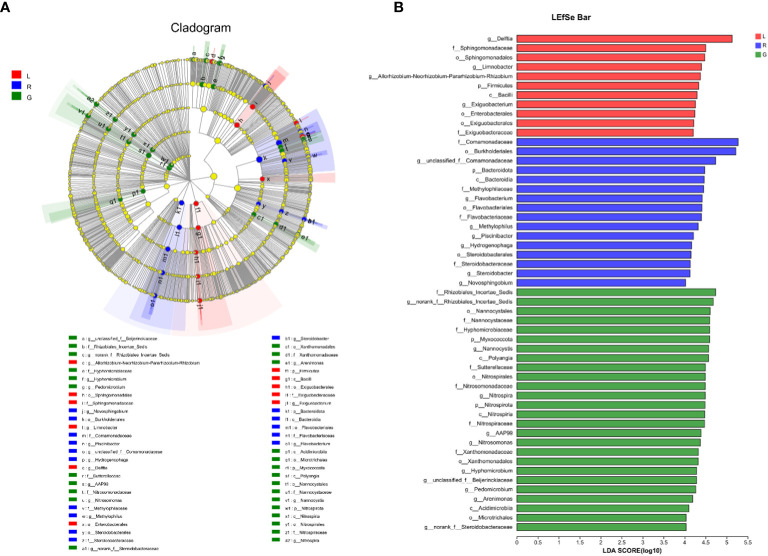
Specific taxa changes in different plant niches using LEfSe analysis. **(A)** LEfSe method was applied to indicate remarkably abundant taxa among root, stem, and leaf groups, as highlighted in the cladogram. The five rings from inside to outside of the cladogram represent phylum, class, order, family and genus. Circles in green, blue and red are differentially abundant taxa identified in three different plant compartments (red = leaf, green = root, blue = stem). **(B)** LDA scores > 4 showed significant group differences in endobacteria at the genus level.

For more details, the taxonomic composition of bacterial species in each plant sample was presented as a heatmap at the genus level based on Bray-curtis distance (**Figure 5**) to visualize the bacterial distribution. The top 50 classified genera belonged to 7 phyla. There were significant different bacterial community structures among plant niches. It can be observed that samples from leaf and stem niches were more relevant. In total, six main groups of niche-specific genera were obtained. Eighteen genera (*Lautropia*, *norank Steroidobacteraceae*, *Ellin6067*, *unclassified Rhizobiales*, *Bacillus*, *norank SC-I-84*, *norank Sutterellaceae*, *Lysobacter*, *Devosia*, *Candidatus Nitrotoga*, *CL500-29 marine group*, *nonrank* Rhizobiales Incertae Sedis, *Nannocystis*, *Nitrospira*, AAP99, *Pedomicrobium*, *Devosia*, and *Arenimonas*) were highly abundant (*P* < 0.05) in root samples; whilst *Methylobacterium-Methylorubrum*, *Limnobacter*, *Exiguobacterium*, *Allorhizobium-Neorhizobium-Pararhizobium-Rhizobium*, and *Nocardioides* were abundant in the leaf samples (*P* < 0.05) and can serve as the best putative indicator bacteria representing the leaf endosphere. In contrast, *Piscinibacter*, *Steroidobacter*, *Methylotenera*, unclassified Sphingomonadaceae, unclassified Comamonadaceae, and *Kineosporia* could be used as putative indicator taxa in the stem endosphere; they were highly abundant in this niche (*P* < 0.05). *Delftia* was the most representative bacterial genus in stem and leaf samples. These findings were further verified by Kruskal-Wallis followed by fdr correction and LEfSe analysis, as shown in the cladogram and the bar figure plotted based on the contributory discriminate taxa with LDA scores > 4 (**Figure 6**). At the genus level, 11, 16, and 26 taxa were significantly enriched in leaf, stem, and root endosphere, respectively (**Figure 6B**), meaning that these species were important in the dissimilation of bacterial community structures among different niches of *E. crassipes*.

In water samples, 3 genera (*hgcI-clade*, *Acinetobacter*, and *CL500-29 marine group*) were predominant, accounting for 45.0% of the high-quality sequences (**Figure S2**). Although most of the dominant genera in specific plant tissues were different, some of the genera were shared among plant and water samples, including the members of *unclassified Camamonadaceae*, *Arenimonas*, *Flavobacterium*, *Exiguobacterium*, *Limnohabitans*, *CL500-29 marine group*, *hglc clade* and so on. This might reflect ecologically coherent networks in water-plant communities.

Taken together, the taxonomic composition of the endobacteriome in *E. crassipes* was influenced by plant compartmentation. Unique taxa were found in each niche. Though the relative abundances often differed among each niche, many genera were shared by different niches, indicating a core bacteriome existed.

### Comparative analyses of bacterial communities

**Figure 7:**
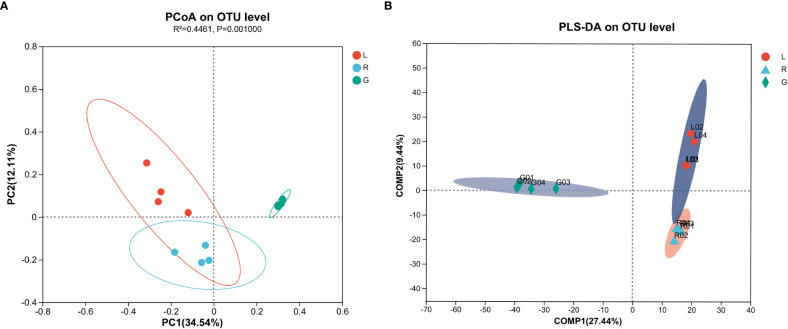
Principal co-ordinates analysis (PCoA) **(A)** and Partial lease squares discriminant analysis (PLS-DA) **(B)** carried out with OUT table based on unweighted UniFrac distances.

To evaluate the degree of differences or similarities of the bacterial communities among the samples from different plant compartments, a beta-diversity analysis based on unweighted UniFrac PCoA (**Figure 7A**) at the OUT level was performed. A scatter plot based on PCoA showed a clear discrimination between the bacteriome in root and other plant niches, which explained 34.5% (axis 1) of the variation. This pattern was recapitulated by hierarchical clustering of Bray-Curtis dissimilarities at OTU level (**Figure S3**). Clustering revealed complete separation of root samples with other plant compartments but stem and leaf samples did not cluster completely, possibly due to their closeness and different growth environment as compared to the root. Then, PLS-DA, a supervised analysis suitable for high-dimensional data, was conducted. The bacterial communities in the samples of root, stem, and leaf groups were strongly separated and clustered into distinct groups, demonstrating the bacterial community structures in these groups were distinctively different (**Figure 7B**). Dissimilarity tests of OTUs based on Bray-Curtis and/or UniFrac distances, including ANOSIM (an analog of univariate ANOVA) and PERMANOVA, were performed (**Table 2**). The calculated *P* values further demonstrated that the bacterial microbiota communities can be remarkably dissimilar for each plant compartment.

**Table 2:** The dissimilarities in *E. crassipes* endophytic bacteriome are niche-dependent at the OUT level.

	ANOSIM		PERMANOVA	
	Statistic	*P*-value	*R^2^*	*P*-value
Bray-curtis	0.7894	0.001^*^	0.6070	0.002^*^
stem versus leaf	0.4063	0.034^*^	0.2989	0.031^*^
stem versus root	1	0.034^*^	0.6947	0.027^*^
root versus leaf	0.9792	0.034^*^	0.5942	0.024^*^
Unweighted UniFrac	0.7477	0.001^*^	N/A	N/A
Weighted UniFrac	0.6458	0.004^*^	N/A	N/A

^*^, Indicates significant differences among groups (P < 0.05). N/A represents not applicable.

### Examination of bacterial interactions

**Figure 8:**
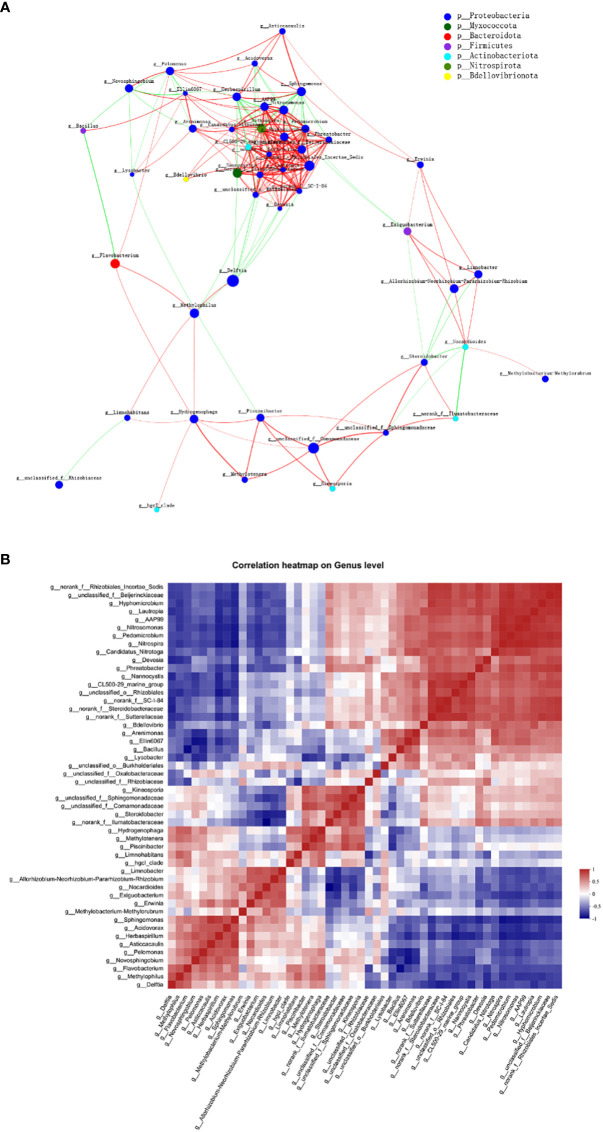
Co-abundance and co-exclusion networks of bacterial communities among three plant niches based on correlation analysis. Each node in network **(A)** represents individual genus and is colored by phylum it belongs to. The connections stand for strong (ρ > 0.6) and significant (*P* < 0.01) Spearman correlations. The node size is proportional to the relative abundance of specific genus in all samples. Lines between nodes show positive (red) or negative (green) connections between nodes. The thickness of each line is proportional to the correlation coefficient value. **(B)** Correlation heatmap analysis of the relationships among the top 50 abundant endophytic bacterial genera. Correlation values ranged from -1 (blue) to 1 (red).

Across all 12 samples from three plant niches, a correlation network analysis was conducted to investigate the co-occurrence interactions among differentially abundant microbes. The correlated genera were assigned to 7 bacterial phyla, among which Proteobacteria made up the largest proportion (77.08%) of all nodes (**Figure 8A**). The average clustering coefficient was 0.61. Though many of the correlations within the community were positive, a reasonable proportion of correlations were negative ones, among 48 genera (ρ > 0.6, *P* < 0.01). Genus *CL500-29 marine group* and *llumatobacteraceae* were negatively associated with *Herbaspirillum* and *Nocardioides*, respectively. *Herbaspirillum*, as well as *Sphingomonas*, conversely, were also negatively associated with *Nitrospira*. Additionally, significant negative correlation was found between *Limnohabitans* and *Rhizobiaceae*, as well as *Bacillus* and *Flavobacterium*; whilst strong positive correlation was observed between *Delftia* and *Methylophilus*.

Interestingly, network analysis at the OUT level showed that OTU1636 and OTU1056 formed only negative connections with other nodes; they were abundant in root and leaf compartments, respectively, and belonged to *Sphingomonas sanguinis* and *Bacillus*, respectively. The hub OTU with the highest degree in the clusters, OTU1756, belonging to *Nitrospira* sp., represented the genus *Nitrospira* in all plant samples; *Nitrospira* was also the hub genus with the most associations in the clusters. OTUs with second highest degree of representation for the genus *Pedomicrobium* were OTU2114 in root and stem and OTU1831 (belonging to *Nitrosomonas*) in all three groups. Based on the betweenness centrality values (Vick-Majors et al., 2014), the top five genera identified were *Flavobacterium*, *Nocardioide*s, norank *Ilumatobacteraceae*, *Devosia*, and *Nitrosomonas*, indicating the prominent roles of these bacteria as putative keystone taxa in the co-occurrence network (Pavlopoulos et al., 2011).

The co-occurrence correlations of endophytic bacterial genera in all plant groups were further investigated in detail as shown in **Figure 8B**. *Nitrospira* and *Nitrosomonas* (ρ = 0.986) as well as unclassified Beijerinckiaceae and norank Rhizobiales Incertae Sedis (ρ = 0.979) were the most positively correlated, respectively, whereas *Nocardioides* and norank *Ilumatobacteraceae* (ρ = –0.888) as well as *Bacillus* and *Flavobacterium* (ρ = –0.855) were the most negatively correlated, respectively.

### Potential functions of bacterial taxa

The organism level bacterial functional traits were calculated with BugBase tool to analyze the effect of niches on the functions and phenotypes of bacteriome (**Figure S4**). Seven phenotypic functional traits were analyzed, including gram positive, gram negative, biofilm forming, pathogenic potential, mobile element containing, oxygen utilizing, and oxidative stress tolerant. In our analysis, a significantly higher abundance of gram-positive (*P* < 0.0001) and biofilm forming bacteria (*P* < 0.0001) was observed in water samples as compared to the plant derived group. On the contrary, a comparatively higher numbers of gram-negative (*P* < 0.0001) and stress tolerance function related (*P* < 0.01) bacteria in plant samples than water samples were observed. A higher proportion of aerobic bacteria and those containing mobile elements in water samples, as compared to root and stem, was observed. In terms of oxygen demand, bacteria from stem showed a higher proportion of facultative anaerobes than those from root and water (*P* < 0.01). The water group showed a higher abundance of anaerobes (*P* < 0.01) than plant groups.

**Figure 9:**
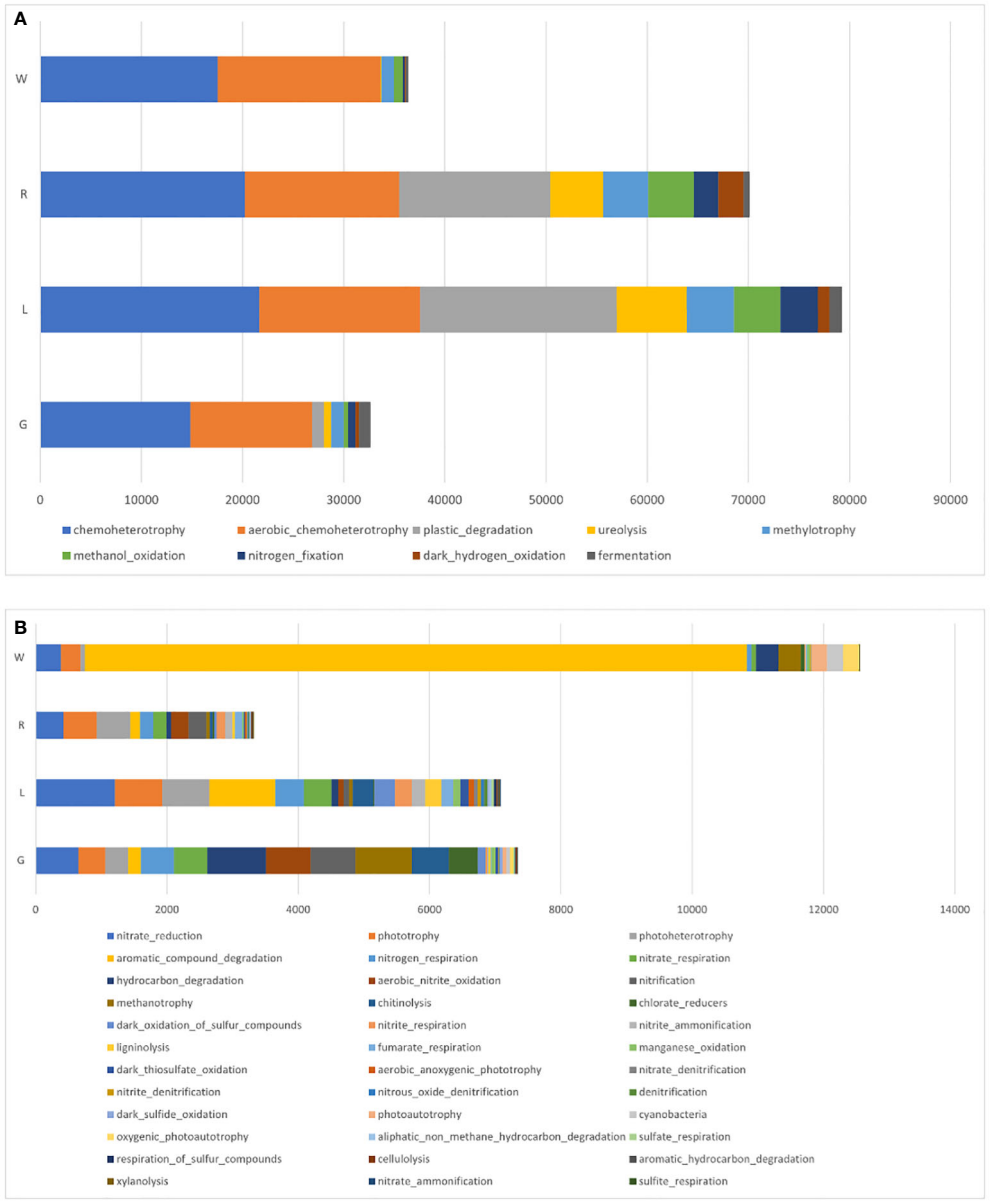
Mean relative abundances of endobacterial functional groups in root, stem, and leaf of *E crassipes*, as well as water samples, via FAPROTAX analysis. **(A)** Major and **(B)** minor functional groups.

FAPROTAX analysis was also applied to infer the presumptive functional content of the bacteria in each plant niche and water (**Figure 9**). Independent of sample origins, the major functions were chemoheterotrophy and aerobic chemoheterotrophy. Functions of nitrate reduction and phototrophy were also assigned to all niches. An increase in OTU’s functional representatives of plastic degradation, ureolysis, methylotrophy, and methanol oxidation was observed in stem and leaf endosphere (**Figure 9A**). By comparison with stem, root and leaf endosphere had a greater abundance of functional content as shown in **Figure 9B**. OTUs involved in aromatic compound degradation was decreased in plant endosphere compared to water. In the root endosphere, the functions were mainly attributed to hydrocarbon degradation, methanotrophy, and chlorate reduction. In contrast, in the leaf samples, OTUs’ functions were largely attributed to ligninolysis, dark thiosulfate oxidation, and denitrification (**Figure 9B**).

### Enumeration and identification of isolated endophytic bacteria in *E. crassipes*

To obtain insight into how the endophytic bacterial populations and diversity differ among microhabitats (root, stem, and leaf) of *E. crassipes*, endophytic bacteria from surface-sterilized vigorous plants (roots, stems, and leaves) were isolated on non-selective media R2A, KB, TSB, 0.1 × LB, and LB. The ddH_2_O used to wash surface-sterilized plant tissues failed to transfer microbial colonies onto agar medium, indicating that the surface sterilization was thorough. The number of CFUs depended on the host niches and ranged from 8.0 × 10^4^ for roots and 4.6 × 10^3^ for stems to 3.1 × 10^3^ for leaves g^-1^ of plant tissue (fresh weight), as expected, the bacterial cell numbers for root were significantly higher (*P* < 0.05) than other parts of the plant. Based on the colony morphological characteristics of isolated strains on agar medium, a total of 55 distinct morphotypes of endophytic bacterial strains were eventually obtained, in which 16 isolates (29.1%) originated from leaves, 8 strains (14.5%) were recovered from stems, and 31 strains (56.4%) were obtained from within root tissues (**Table S2**).

Based on the analyses of 16 S rRNA gene sequences, all isolated bacteria were divided into four phyla: Proteobacteria (30.9%), Firmicutes (58.2%), Bacteroidetes (3.6%) and Actinobacteria (7.3%). These isolated endophytes were then be assigned to 14 families and 20 genera. Comparative analysis of OTUs with cultured bacterial strains showed consistent association of 14 out of these 20 cultured bacterial genera that validated our 16S rRNA gene analysis (**Table S3**). Bacillus (54.5% of the total isolates) was the predominant genus in all three niches, while most of the other genera, such as Stenotrophomonas, Cytophaga, Herbaspirillum, Roseateles, Zavarzinia and Xanthobacter were niche-specific and isolated rarely, represented by only one strain (**Table S2**). Rhizobium and Acinetobacter were the second most widely dispersed genera and were isolated from root and leaf tissues, respectively. Phylogenetic analyses revealed that most bacterial isolates shared high sequence similarities (98.25 to 99.93%) with recognized species. Strains EGA240408 and EGA240374 exhibited 94.65 and 96.27% 16S rRNA gene sequence homology, strongly indicating that these two strains could be new members of the genera *Zavarzinia* and *Roseomonas*, respectively.

### *In vitro* functional characterization of isolated endophytes

We hypothesized that *E. crassipes* endobacteria could manifest their beneficial effects on plant physiology which might be important in agricultural practices. Thus, all 55 isolated endobacterial strains were examined for several traits that are often associated with plant defense and growth promotion via different bioassays (**Table S4**). Most of the *Bacillus* isolates from different plant niches displayed protease, chitinase and cellulase activity and were able to produce IAA. Mineral phosphate solubilization activity and ACC deaminase were also detected in many of these isolates (14 and 12, respectively). All *Acinetobacter* isolates (3) originated from leaf showed abilities of organic phosphate solubilization and siderophore production. *Pseudomonas* strains possessed the highest number (8 out of 9) of the recognized PGP traits tested. All three *Rhizobium* isolates displayed nitrogen fixation and mineral phosphate solubilization ability. *Sphingomonas* (2) were able to solubilize both inorganic and organic phosphate. Moreover, *Roseateles depolymerans* was shown to be able to degrade biodegradable plastics, i.e., poly(butylene succinate)-*co*-(butylene adipate) (PBSA), while isolates of *Roseomonas* and *Zavarzinia* spp. could be resistant to to As(III) up to 0.5 mM. All the other isolates exert different patterns of PGP profiles. In summary, *E. crassipes* is a good reservoir of endobacteria that is promising for exploration of potential plant growth beneficial bacteria.

## Discussion

The phytomicrobiome is a group of microorganisms that associate and interact with its host plant; the host plus its phytomicrobiome, including bacteria, archaea, fungi, viruses, algae etc., collectively constitute a holobiont (Lyu et al., 2021b). Plant-associated endobacteria, which have been studied over the past nine decades, play important roles in endowing hosts with the ability to adapt to environment that might be liable for growth and resistance of host plants to stresses (Lyu et al., 2021a). *E. crassipes* is a floating plant that has attracted attention with regard to phytoremediation and N and P recycling manifested by bacteria isolated from it. There have been some studies on the identification of rhizosphere microorganisms (Fu and Zheng, 2016; Rajagopal et al., 2020) and whole-plant endobacteria in *E. crassipes* (Lan et al., 2008). Scant data are available regarding bacteriome structural endobacteria of *E. crassipes*. To extend our knowledge of bacterial diversity related to aquatic macrophytes, in this study, we investigated the structural diversity and niche differentiation of bacteria present in the root, shoot and leaf endospheres of wild-growing *E. crassipes*, using a culture-dependent approach and culture-independent molecular techniques. Although the role of host filtering of endophytes was carefully considered (Bai et al., 2015; Ndour et al., 2017), research regarding the association of water bacteria with the plant root (vertical transmission) of *E. crassipes* is lacking. Therefore, the dynamic assembly of bacteriome of *E. crassipes* in the water-root continuum is discussed.

In the present study, we isolated 55 endobacterial strains from root, stem, and leaf parts comprising 20 genera in 4 bacterial phyla: Proteobacteria, Firmicutes, Bacteroidetes and Actinobacteria. These phyla are commonly identified as endophytes by culture-dependent isolation (Santoyo et al., 2016). The dominant endobacterial genera (*Bacillus*, *Rhizobium*, and *Acinetobacter*) obtained in this study were different from those of a related study, in which *Microbacterium*, *Pseudomonas*, and *Bacillus* were the most abundant three genera of endobacteria isolated from *E. crassipes* whole plants (Lan et al., 2008). Moreover, Lan et al. (2008) obtained more endophytic genera (32) than we did (20). The apparent differences in bacterial composition can be attributed to water and atmospheric environmental conditions (O'Brien et al., 2020), plant growth stage, and the number of culture media evaluated: 20 in Lan et al. (2008) versus 5 in our study. In the present study, some of the isolates diminished during purification cycles on agar plates, signifying that there were some endophytes with either key plant dependencies or require specific media to persist and thrive (Oberhardt et al., 2015; Lewis et al., 2021).

Culture-independent characterization of bacterial communities was conducted to facilitate our understanding of the bacterial assembly in *E. crassipes*. The sampled endosphere in the present study was inhabited by diverse bacterial communities. There were significant differences in bacterial diversity among plant tissues of *E. crassipes*, as demonstrated by rarefaction curves and alpha-diversity indexes. No significant difference was observed between means of the alpha-diversity indexes of stems versus leaves. A similar result was reported for *Macleaya cordata*, for which stem and leaf niches were alike in endobacterial diversity (Lei et al., 2021). The significant (*P* < 0.05) sequential decline of endobacterial diversity and evenness from roots to stems and leaves was interpreted to indicate the strong competition between rhizobacteria as niches become more defined, leading to a limited number of bacteria surviving, or possibly that some endophytic bacteria have tissue-specific preferences within the host (Diwan et al., 2022), leading to occurrence of a niche-oriented filtration process. This result was consistent with many other studies showing that roots had the highest bacterial diversity (Wang et al., 2019; Alibrandi et al., 2020).

Our results showed that the bacterial community assembly and final composition of plant endosphere and water were different. In general, the dominant bacterial phyla in the experimental samples were Proteobacteria, Actinobacteriota, and Bacteroidota. Endobacterial species belonging to these three major phyla were reported as dominate bacterial microbiota in terrestrial (Wang et al., 2020) and aquatic plants (Wang et al., 2023). The abundance of Proteobacteria in the endospheres of *E. crassipes* was increased when compared to water, which can be attributed to the accessibility of reduced-carbon sources (Peiffer et al., 2013), where root cells can secrete plant derived compounds that induce chemotaxis (Bulgarelli et al., 2013; Lyu et al., 2021a). Relatively high levels of bacterial species from Actinobacteriota in all samples, especially in water, was also in line with the findings in Wang et al. (2023), who indicated the pivotal roles of members of Actinobacteriota in protecting host plants from pathogens by the secretion of antimicrobial compounds (Bulgarelli et al., 2013) and in enhanced biological phosphorus removal (EBPR) systems (Liu et al., 2019a). In the current experiment, Verrucomicrobiot were abundant in water samples while Nitrospirota were abundant in root samples. Verrucomicrobiota and Nitrospirota can biosynthesize secondary-metabolites, with high numbers of terpene clusters (Nayfach et al., 2021). Myxococcota was found in relatively high abundance in root tissues in the current study and in sludge (Gu et al., 2022), which indicated that the bacterial species in the Myxococcota phylum can contribute to fermentation and reduction of dissimilatory nitrate and sulfate (Murphy et al., 2021).

To elucidate the host plant compartments selection of endophytic bacterial community compositions, a ternary plot was generated based on the mean relative abundance of each genus in each plant niche (root, stem, and leaf). Ternary plotting visualized the restrictive selection of endophytic bacterial community by roots, compared to other plant compartments (stem tissue). Root tissue selected members of the families Nannocystaceae and Sutterellaceae, whilst the leaf harbored a high abundance of Rhizobiaceae and Sphingomonadaceae. Sphingomonadaceae and Rhizobiaceae were also the most common families occurring in the *Arabidopsis thaliana* phyllosphere (Vorholt, 2012) and many other plant species. Strains from Sphingomonadaceae and Rhizobiaceae facilitate nutrient sharing, and metabolic shifts contributed to co-colonization by them of the oligotrophic discrete habitat represented by the leaf (Hemmerle et al., 2022). Extensive studies of nitrogen fixation by Rhizobiaceae was motivated by economics and agriculture (Ludwig, 1980; Yang et al., 2020). Rhizobiaceae can facilitate plant development by atmospheric nitrogen fixation or by the alleviation of nutrient limitation (Lyu et al., 2021a) by the allocation of proteome resources to fatty acid beta-oxidation (Hemmerle et al., 2022). The family Sphingomonadaceae can use a wide range of plant-originated substrates for phyllospheric colonization across a range of plant species (Vorholt, 2012) and was shown to protect *A. thaliana* from bacterial pathogens (Innerebner et al., 2011). The family Nannocystaceae (phylum Myxococcota) that contains the genus *Nannocystis*, one of the most abundant genera in roots of *E. crassipes*, might play a role in suppressing niche competitors and is a rich source for diverse natural products (Herrmann et al., 2017) that can be used for drug development (Liao et al., 2016). Sutterellaceae from roots of *E. crassipes* is a family of the Betaproteobacteria (order Burkholderiales) that were previously mainly found as intestinal microbiota (Morotomi et al., 2011). The members in this family were found to be correlated with human diseases or can be used in disease control (Lin et al., 2020). Endophytes can be transported from roots (Chi et al., 2005) to the plant tissues above the water surface, stems and leaves in this case and from the adjacent atmosphere, that is, by horizontal dispersal (Zhou et al., 2020). Some of the endobacterial families that were present in the stem were differentially selected by the root and leaf. Venn diagram analysis showed that some bacteria in the stem did not drift toward the leaf, which we attributed to differences in microbial flora and nutrient sources, or other environmental factors.

The results of beta-diversity analyses indicated that plant compartments affect endobacterial community composition. PCoA and PLS-DA diagrams showed separations in the endobacterial community structure among plant niches at the OTU level, which indicated significant differences in endobacteria in the respective plant compartments. These results were supported by heatmapping of genera among endosphere niches, and an indicator taxa analysis. Several genera, most of which belong to Proteobacteria, contribute to the observed differences in the endobacterial profiles among plant niches, such as *Methylobacterium-Methylorubrum*, *Limnobacter*, *Exiguobacterium*, and *Allorhizobium-Neorhizobium-Pararhizobium-Rhizobium* in leaf (*P* < 0.05), *Piscinibacter* and *Steroidobacter* in the stem (*P* < 0.01), and *Nannocystis*, *Nitrospira*, *Rhizobiales Incertae Sedis*, and *AAP99* in root tissues (*P* < 0.01). *Rhizobiales Incertae Sedis* can utilize a wide range of carbohydrates and facilitate nitrogen fixation (Liu et al., 2022); thus, its high abundance in roots could be related to symbiotic nitrogen fixation in the root systems of *E. crassipes*. Aerobic anoxygenic phototrophic (AAP) bacteria are commonly known to inhabit limnic and marine, as well as terrestrial environments (Csotonyi et al., 2010; Qiu et al., 2019) and play unique roles in biogeochemical cycles. Some of them are capable of degrading pollutants, such as organic matter, to acquire energy (Hiraishi et al., 2002). The relative abundance of the genus *AAP99* was shown to increase significantly after UV irradiation of wastewater (Hao et al., 2022). *Nitrospira*, an aerobic chemolithoautotroph, is adapted to oligotrophic conditions and plays a pivotal role in nitrification in terrestrial and aquatic ecosystems. Some *Nitrospira* members are complete nitrifiers (Kits et al., 2017; Vijayan et al., 2021), and thus are critical for the nitrogen cycling process. *Piscinibacter* has been widely isolated from eutrophic aquatic environments. Species in the genus *Piscinibacter* are methylotrophic bacteria that participate in the soil carbon cycle (Kalyuzhnaya et al., 2008). This genus was one of the dominant bacterial genera in the rhizospheric soil of *Fritillaria taipaiensis* (Zhou et al., 2022) and was also reported to dominate in the endophytic bacteria from fruit of *Morinda citrifolia* (Cao et al., 2014). Moreover, a *Piscinibacter* species isolated from a paddy field ecosystem exerted an antagonistic effect on *Magnaporthe oryzae* (Wei et al., 2021)*. Steroidobacter* were previously isolated from sludge, and reported as steroidal hormone decomposers (Fahrbach et al., 2008). Some members of *Steroidobacter* could degrade polysaccharides and even agar (Sakai et al., 2014). The genus *Steroidobacter* was also abundant in the endo-fibril tissues of *Panax notoginseng* roots (Dong et al., 2018); the result was similar with our study showing that *Steroidobacter* highly dominated in the fibrous stem of *E. crassipes*. Microbial colonization ability is related to its adaptation to the host’s internal environment and the availability and utilization of substrates therein. Since leaves are exposed to various and often challenging environments, i.e., desiccation, UV irradiation, and nutrient stress, special microorganisms often thrive in this habitat. Methylobacterium predominated in the endophytic bacterial communities of *E. crassipes* leaf. They are within the order Rhizobiales and are facultative methylotrophs, thus are type microbes for methanol assimilation metabolism. They are ubiquitously present in a wide range of environments and were shown to dominate the leaf interior of *Macleaya cordata* (Lei et al., 2021), rely on methanol released by host plants (Galbally and Kirstine, 2002), and mitigated the impact of water limitation in *Lens culinaris* Medic (Jorge et al., 2019), as well as improved potato yield under salinity stress (Grossi et al., 2020). Moreover, strains from *Methylobacterium-Methylorubrum* were isolated from inside the International Space Station, exerting plant growth promotion effects under microgravity conditions (Bijlani et al., 2021). Members of the *Limnobacter* genus were detected in a wide ranges of environments, i.e., seawater, human intestines (Rigsbee et al., 2011) and volcanic deposits and were associated with degradation of aromatic compounds (Vedler et al., 2013). This genus was also one of the most prevalent air bacterial genera tolerating extreme environments (Cai et al., 2022). A recent study demonstrated *Limnobacter* was abundant in the capsule of *Serapias vomeracea* (Alibrandi et al., 2020) and in the phyllosphere of *Nicotiana tabacum* (Xing et al., 2021). *Exiguobacterium* has been reported to contribute to plant growth and health, probably due to its broad-spectrum antagonistic effects on phytopathogens and the abilities to fix atmospheric nitrogen and solubilize phosphate (Dastager et al., 2010; Ji-Youn et al., 2020). It is interesting to note that the abundance of *Exiguobacterium* in rice leaves was greatly improved with the application of rice straw and NPK fertilizer in the soil (Jin et al., 2023). In our study, *Limnobacter* and *Exiguobacterium* strongly dominated in the *E. crassipes* leaf endosphere, indicating that these two genera tend to colonize preferentially the above-water surface parts of *E. crassipes*. The *Allorhizobium-Neorhizobium-Pararhizobium-Rhizobium* clade dominated in leaf endosphere of *E. crassipes*, which concurred with leaf-findings regarding *Dioscorea alata* (Kihara et al., 2022) and two rice genotypes (Pusa Basmati-1 and BPT-5204) (Kumar et al., 2021). Interestingly, this clade was increased in response to the exposure to pollutants, such as plasticizers (Bai et al., 2020), cadmium (Guo and Chi, 2014), and waste water (Bigott et al., 2022). Thus, this plant growth-promoting clade might be resistant against various abiotic stresses and outcompete other bacteria inside plant tissues, such as *E. crassipes* leaf (in this study) as well as lettuce roots (Bigott et al., 2022). As in water, the abundance for CL500-29 marine group, *Acinetobacter* and hgcI-clade (*Cyanobacteria*) were significantly higher in comparison with plant groups. Similar results were obtained from static water in ecological floating beds (EFBs), showing that *Cyanobacteria* and *CL500-29 marine group* are dominant taxa (Zhang et al., 2022). The thriving of *Cyanobacteria* indicated a certain degree of eutrophication of the lake water. Bai et al. (2015) and Lei et al. (2021) reported the root endomicrobiome structures were significantly different from those in stem and leaf. Thus, the data from present study and former research efforts indicate that plant compartments are one of the driving forces of endobacterial assembly. The data presented here suggested that *E. crassipes* compartments drive the enrichment of different endobacterial taxa to at least some extent. These differences are probably attributed to the vagaries of factors such as nutrient availabilities, physiology of plant tissues, environmental factors, microbe-microbe interactions, etc. (Hunter et al., 2010; Kumar et al., 2019). Moreover, bacterial functions have been proposed to be key to their recruitment by hosts (Burke et al., 2011).

As to the co-occurrence patterns of bacteria in different parts of *E. crassipes*, *Flavobacterium*, *Nocardioide*s, norank *Ilumatobacteraceae*, *Devosia*, and *Nitrosomonas* were the top five genera based on the betweenness centrality values, indicating they were important nodes in the co-occurrence network. *Nocardioide*s species have been isolated from various sources, such as soil, water (Park et al., 2020) and plants (Liu et al., 2019b). Members of this genus function in the degradation of organic pollutants (Topp et al., 2000) and alleviation of salt stress in wheat seedlings (Meena et al., 2020). *Nitrosomonas* is an ammonia-oxidizing bacteria and participates in a crucial step of the N cycle (Otaka et al., 2022). Though many of the strains in *Flavobacterium* are human pathogens, members of this genus also exert beneficial effects on plant growth (Manter et al., 2010) and pathogen resistance (Kolton et al., 2014). Moreover, *Flavobacterium* was found to have more connections with other taxa at the seedling stage for *Typha orientalis* roots (Wang et al., 2023). *Devosia* is a symbiotic nitrogen-fixing bacterium (Bautista et al., 2010) and was identified as a keystone genus in previous research (Ji et al., 2022). Thus, future work is needed to better understand the roles of *Devosia* and *Flavobacterium* in co-occurrence networks. Based on the co-occurrence correlation analysis, many of the positive correlations in our study were between different phyla, indicating that metabolic cooperation might be important in shaping taxa co-occurrence (Zelezniak et al., 2015). Moreover, co-occurrences between genera are influenced by *E. crassipes* compartments, indicating that besides phenotypic plasticity (Hemmerle et al., 2022), niche overlap of bacteria could lead to positive correlations, which is consistent with previous research (Deng et al., 2019).

The possible functions of the endobacterial community in *E. crassipes* was predicted using FAPROTAX, which is based on the current prokaryotic function annotation library of cultivable bacteria. Our study showed major assignments of functions to chemoheterotrophy, aerobic chemoheterotrophy, plastic degradation, and ureolysis. To some degree, functions were generally related to phototrophy, photoheterotrophy, nitrogen cycling, and hydrocarbon degradation. Root extracts play pivotal roles in plant-bacteria integration. The functions in phototrophy and photoheterotrophy were increased in plant-associated bacteria in our study; this is partially supported by a previous study showing that crude exudates of *E. crassipes* promoted the growth of photosynthetic bacteria (Chang et al., 2007). There have been some previous functional studies in bacteria associated with wetland plants. A recent work based on 16S rRNA gene sequencing revealed a high proportion of functions related to chemoheterotrophy, aerobic chemoheterotrophy, fermentation, and nitrate reduction in the root endosphere of *T. orientalis* (Wang et al., 2023). Based on BugBase analysis, *E. crassipes* hosted a high level of bacterial active in stress-tolerance, in comparison with adjacent water, indicating that the host plant recruited more bacteria for stress defense. However, these results were predictive, thus the functionality of bacteria must be determined by metagenomic studies and *in situ* experimental settings.

Conventional culture-dependent methods were applied for culturable endobacteriome analysis in *E. crassipes*, culminating in the isolation of diverse bacterial isolates. The endogenous bacterial counts obtained from leaf, stem, and roots of *E. crassipes* were comparable with that of (Chen et al., 2012), within the range of 10^3^ to 10^4^ CFU g^-1^ FW. The diversity of isolates obtained did not mirror that of the respective bacteriome. *Delftia* being an abundant member in endobacteriome that was missing in our isolated collections. This might be due to a reduced cultivability under the cultivation environment being used. Many of the endophytic bacterial isolates possessed ACC deaminase activity, meaning that they might reduce pollutant toxicity and improve plant growth (Afzal et al., 2014). Isolates affiliated with *Bacillus* were metabolically versatile, exhibiting a wide variety of activities such as compound degradation and nutrient cycling, comparable to that from a previous study (Fan and Smith, 2021). Some *Bacillus* and *Pseudomonas* species also exerted biocontrol abilities (Santoyo et al., 2012). *Roseateles depolymerans* and isolates of *Roseomonas* and *Zavarzinia* spp. were shown to degrade biodegradable plastics and resist to heavy metal (As), respectively, which were in accordance with previous reports (Shah et al., 2014; Sun et al., 2020). The re-inoculation of strains (*Bacillus* and *Aeromonas* sp.) isolated from *E. crassipes* facilitated in the removal of heavy metals by *E. crassipes* (Kabeer et al., 2022). Based on the high-throughput analysis, the components of culture media could be optimized accordingly (Dedysh, 2011), specifically for slow-growing, oligotrophic microorganisms that are difficult to manipulate in the laboratory, in order to isolate more functional bacterial strains with potential values. Above all, the isolates from this study showed versatile metabolic capabilities, thus making them valuable resources in terms of sustainable utilization for biosynthesis of specific materials (Biedendieck et al., 2021), plant growth promotion, and bioremediation technologies.

## Conclusions

As far as is known, this study provided the first in-depth analysis of endobacterial diversity and assemblies of different compartments in wild-grown water hyacinth (*Eichhornia crassipes*), using next-generation sequencing technology. We found that endobacterial community structures significantly changed among root, stem, and leaves of *E. crassipes*. The systematic changes in endobacterial colonization of *E. crassipes*, with a reduction in diversity from root to leaf, indicated an active role of the plant in endobacteriome selection. The potential effect of endobacteria on bioremediation and plant growth and health, based on *in vitro* functional characterization of bacterial strains, further supports the evidence of the beneficial roles of water hyacinth endobacteria for the water hyacinth host. This study provides a new insight into the complexities and interactions of endobacterial community of *E. crassipes*, opening new unexplored scenarios for water hyacinth holobiont studies. This work also guides the increase of resource utilization of *E. crassipes*, since this aquatic plant species represents a rich reservoir of bacterial strains that are promising for exploration as potential plant growth promoting bacteria or bioremediators for more climate change resilient agricultural and environmental practices.

## Data availability statement

Sequencing data has been deposited in the NCBI Sequence Read Archive (SRA) under the BioProject accession number PRJNA947841.

## Author contributions

DF: conceptualization, methodology, supervision, and funding acquisition. DF, TS, SL, and OX: writing – original draft, formal analysis and visualization. DF, SL, and OX: investigation. CG and QC: resources. DS: writing – review and editing. All authors contributed to the article and approved the submitted version.

## Supplementary material

Supplementary Figure 1 Analysis of bacterial communities at family level in water where *E. crassipes* grew.

Supplementary Figure 2 The bacterial community of lake water based on Circos diagram. Less abundant (*P* < 0.01) phyla are summarized as “others”.

Supplementary Figure 3 Clustering analysis of the endobacterial communities in different compartments based on OTU abundance-based Bray-Curtis distances. R, stem; L, leaf; G, root.

Supplementary Figure 4 BugBase showing differences in niche-associated bacteriome.
