# Supplementary figures and images for: Characterization of endophytic bacteriome diversity and associated beneficial bacteria inhabiting a macrophyte Eichhornia crassipes
Source: Front Plant Sci. 2023 Jun 19;14:1176648. doi: 10.3389/fpls.2023.1176648 (PMC10316030; doi:10.3389/fpls.2023.1176648)

# Community barplot analysis

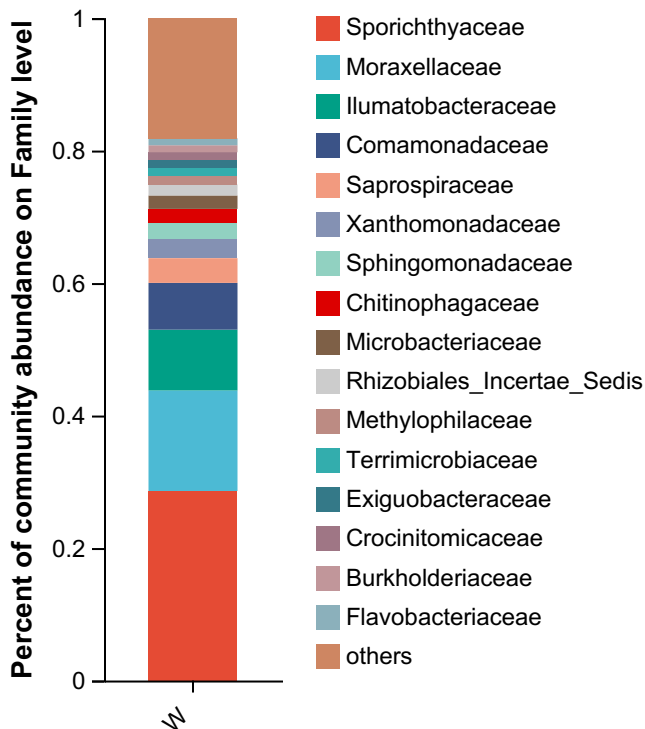

Supplement: Supplementary Figure 1 — Analysis of bacterial communities at family level in water where E. crassipes grew. [file DataSheet_1.pdf]

# Circos

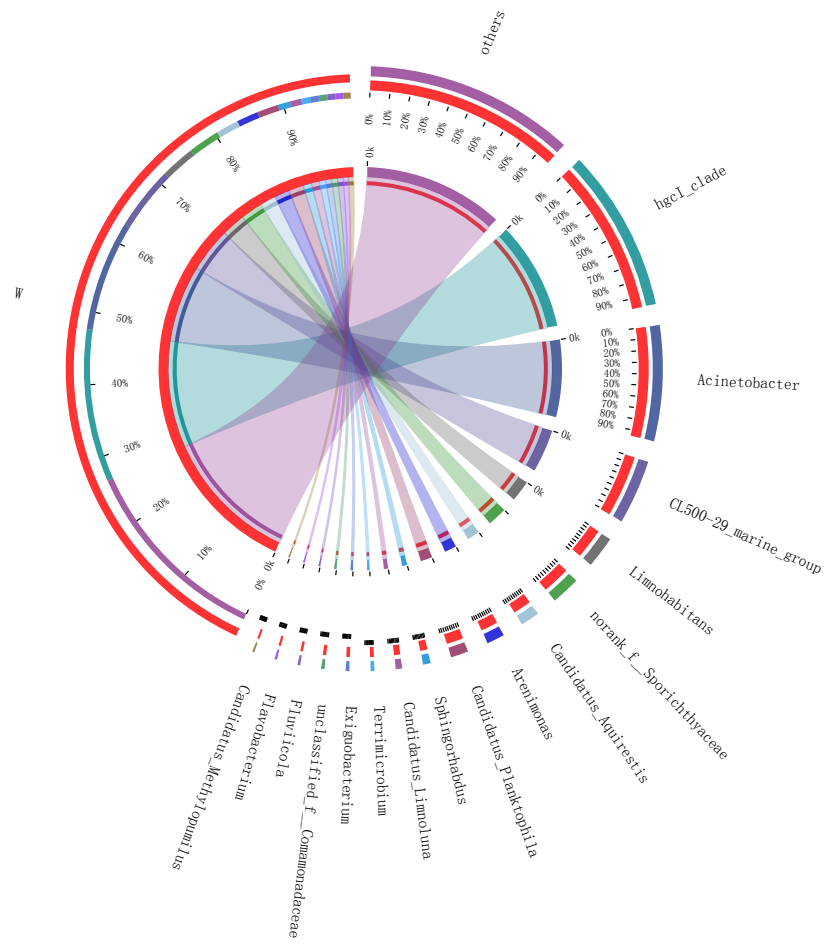

Supplement: Supplementary Figure 2 — The bacterial community of lake water based on Circos diagram. Less abundant (P < 0.01) phyla are summarized as “others”. [file DataSheet_2.pdf]

# Hierarchical clustering tree on OTU level

Group

L

R

G

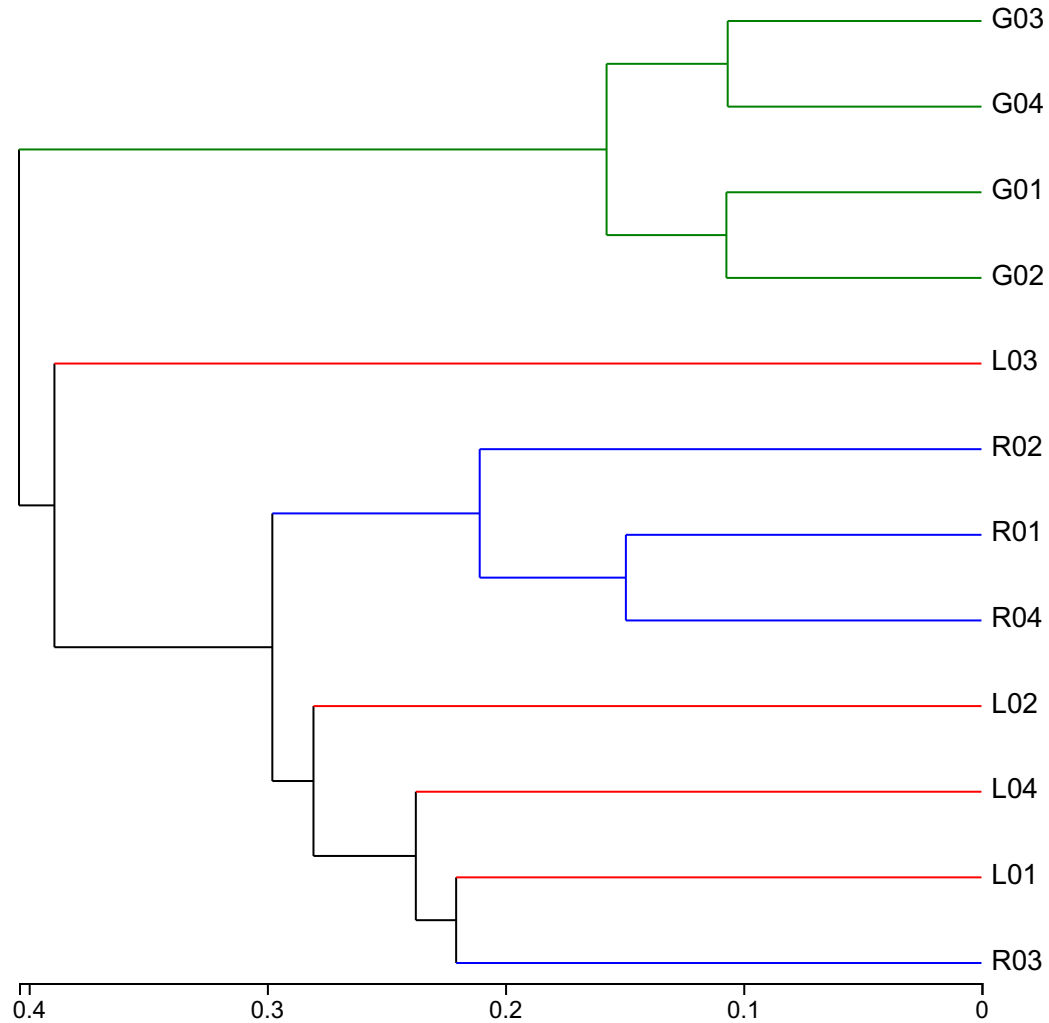

Supplement: Supplementary Figure 3 — Clustering analysis of the endobacterial communities in different compartments based on OTU abundance-based Bray-Curtis distances. R, stem; L, leaf; G, root. [file DataSheet_3.pdf]

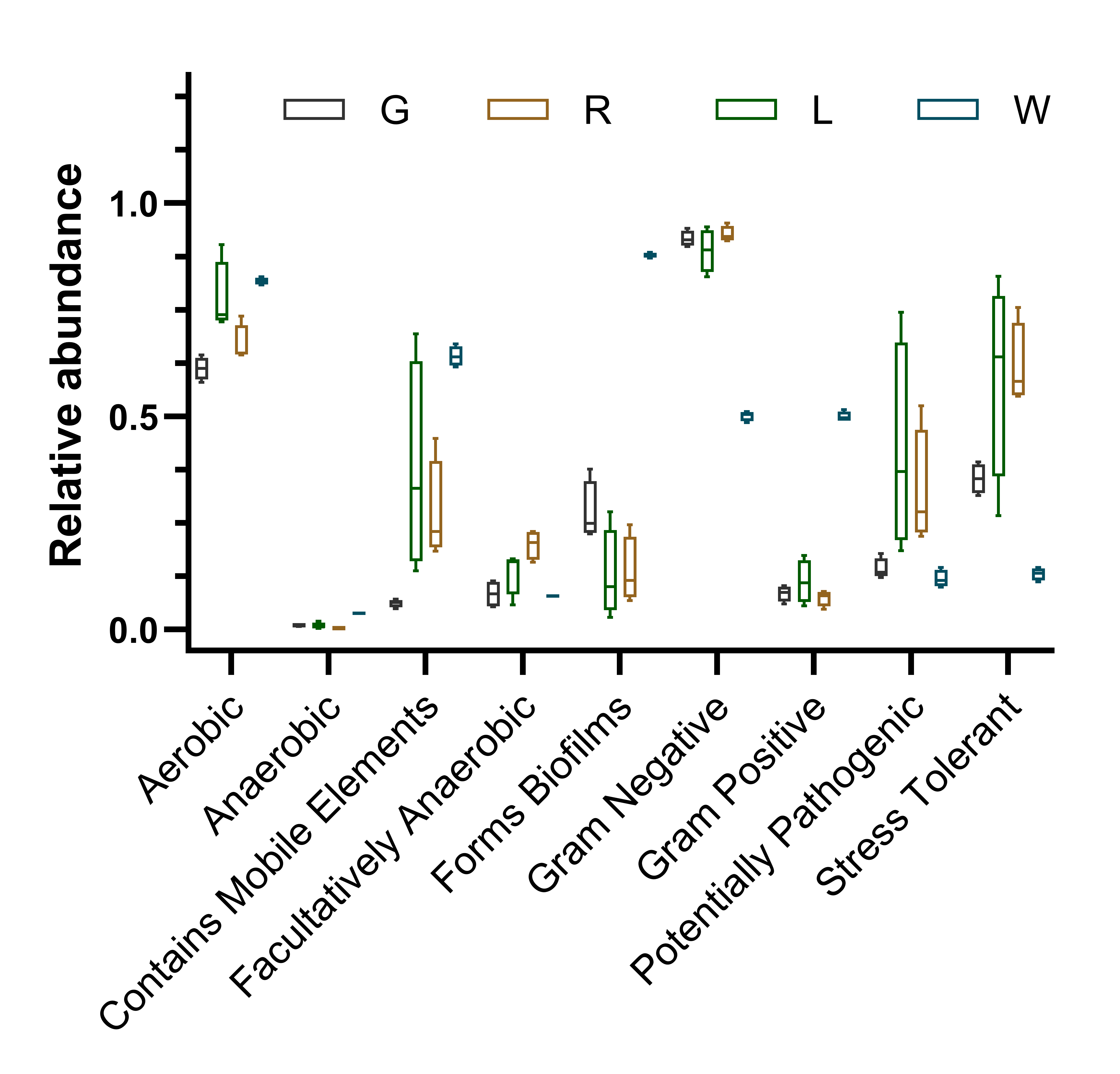

Supplement: Supplementary Figure 4 — BugBase showing differences in niche-associated bacteriome. [file Image_1.tif]
