# Supplementary material for: Characterization of endophytic bacteriome diversity and associated beneficial bacteria inhabiting a macrophyte Eichhornia crassipes
Source: Front Plant Sci. 2023 Jun 19;14:1176648. doi: 10.3389/fpls.2023.1176648 (PMC10316030; doi:10.3389/fpls.2023.1176648)
Supplement: Supplementary file 5 [file Table_1.docx]

| **Table S1.** | | |  | Physicochemical parameters of lake water. | | | | | | | | | | | | | | | | | | | | |  |  |  |  |  |
| --- | --- | --- | --- | --- | --- | --- | --- | --- | --- | --- | --- | --- | --- | --- | --- | --- | --- | --- | --- | --- | --- | --- | --- | --- | --- | --- | --- | --- | --- |
| **pH** | **NH_3_-N** | | | | | **TP** | **COD** | **BOD_5_** | **TOC** | **nitrates** | | **nitrite** | **sulfates** | **K** | **Fe** | **Na** | | **Cd** | **Pb** | | **Cr** | **As** | | **Ni** | | **Ca** | | **Mg** | **TDS** |
|  | mg/L | | | | | | | | | | | | | | | | | | | | | | | | | | | | |
| 8.1 | 0.142 | | | | | 0.02 | 11 | 2.3 | 7.7 | 0.805 | | - | 20.9 | 3.15 | 0.0240 | 18.6 | | - | - | | - | 0.00157 | | 0.00160 | | 37.0 | | 8.57 | 71 |
|  | | |  | -, means under limit of detection | | | | | | | | | | | | | | | | | | | | |  |  |  |  |  |
|  | | |  |  | | | | | | | | | | | | | | | | | | | | |  |  |  |  |  |
| **Table S2.** | | |  | Molecular identification and functional profile of culturable endobacterial isolates using 16S rRNA as query sequences. | | | | | | | | | | | | | | | | | | | | |  |  |  |  |  |
| **Origin** | | **Isolate name** | | | **Closet relative (NCBI)** | | | | | | **Closest type strain (accession number)** | | | | | | **%Similarity (EzTaxon)** | | | **Phylum** | | | **Family** | | | |  |  |  |
| leaf | | EGA240353 | | | *Bacillus subtilis* | | | | | | *Bacillus tequilensis* (AYTO01000043) | | | | | | 99.58 | | | Firmicutes | | | Bacillaceae | | | |  |  |  |
|  |  | EGA240354 | | | *Bacillus subtilis* | | | | | | *Bacillus tequilensis* (AYTO01000043) | | | | | | 99.86 | | | Firmicutes | | | Bacillaceae | | | |  |  |  |
|  |  | EGA240355 | | | *Bacillus subtilis* | | | | | | *Bacillus tequilensis* (AYTO01000043) | | | | | | 99.86 | | | Firmicutes | | | Bacillaceae | | | |  |  |  |
|  |  | EGA240356 | | | *Bacillus subtilis* | | | | | | *Bacillus tequilensis* (AYTO01000043) | | | | | | 99.51 | | | Firmicutes | | | Bacillaceae | | | |  |  |  |
|  |  | EGA240357 | | | *Cytophaga* sp. VM1T | | | | | | *Cytophaga massiliensis* (EF394924) | | | | | | 99.21 | | | Bacteroidetes | | | Cytophagaceae | | | |  |  |  |
|  |  | EGA240358 | | | *Roseateles depolymerans* | | | | | | *Roseateles depolymerans* (CP013729) | | | | | | 99.72 | | | Proteobacteria | | | norank | | | |  |  |  |
|  |  | EGA240359 | | | *Herbaspirillum huttiense* | | | | | | *Herbaspirillum aquaticum* (NJGV01000018) | | | | | | 99.93 | | | Proteobacteria | | | Oxalobacteraceae | | | |  |  |  |
|  |  | EGA240360 | | | *Bacillus subtilis* | | | | | | *Bacillus tequilensis* (AYTO01000043) | | | | | | 99.58 | | | Firmicutes | | | Bacillaceae | | | |  |  |  |
|  |  | EGA240361 | | | *Acinetobacter soli* | | | | | | *Acinetobacter soli* (APPU01000012) | | | | | | 99.30 | | | Proteobacteria | | | Moraxellaceae | | | |  |  |  |
|  |  | EGA240363 | | | *Acinetobacter* sp. | | | | | | *Acinetobacter seifertii* (KB851199) | | | | | | 99.15 | | | Proteobacteria | | | Moraxellaceae | | | |  |  |  |
|  |  | EGA240364 | | | *Acinetobacter seifertii* | | | | | | *Acinetobacter seifertii* (KB851199) | | | | | | 99.21 | | | Proteobacteria | | | Moraxellaceae | | | |  |  |  |
|  |  | EGA240365 | | | *Agrobacterium vitis* | | | | | | CP000633_s (CP000633) | | | | | | 98.90 | | | Proteobacteria | | | Rhizobiaceae | | | |  |  |  |
|  |  | EGA240366 | | | *Sphingomonas azotifigens* | | | | | | *Sphingomonas azotifigens* (BCTR01000108) | | | | | | 99.49 | | | Proteobacteria | | | Sphingomonadaceae | | | |  |  |  |
|  |  | EGA240367 | | | *Bacillus subtilis* | | | | | | *Bacillus tequilensis* (AYTO01000043) | | | | | | 99.44 | | | Firmicutes | | | Bacillaceae | | | |  |  |  |
|  |  | EGA240368 | | | *Sphingomonas trueperi* | | | | | | *Sphingomonas trueperi* (X97776) | | | | | | 99.56 | | | Proteobacteria | | | Sphingomonadaceae | | | |  |  |  |
|  |  | EGA240369 | | | *Microbacterium* sp HBUM179310 | | | | | | *Microbacterium hydrothermale* (HM222660) | | | | | | 98.71 | | | Actinobacteria | | | Microbacteriaceae | | | |  |  |  |
| stem | | EGA240370 | | | *Bacillus subtilis* | | | | | | *Bacillus tequilensis* (AYTO01000043) | | | | | | 99.58 | | | Firmicutes | | | Bacillaceae | | | |  |  |  |
|  |  | EGA240371 | | | *Bacillus subtilis* | | | | | | *Bacillus tequilensis* (AYTO01000043) | | | | | | 99.93 | | | Firmicutes | | | Bacillaceae | | | |  |  |  |
|  |  | EGA240372 | | | *Bacillus subtilis* | | | | | | *Bacillus tequilensis* (AYTO01000043) | | | | | | 99.51 | | | Firmicutes | | | Bacillaceae | | | |  |  |  |
|  |  | EGA240373 | | | *Bacillus subtilis* | | | | | | *Bacillus tequilensis* (AYTO01000043) | | | | | | 99.58 | | | Firmicutes | | | Bacillaceae | | | |  |  |  |
|  |  | EGA240374 | | | *Zavarzinia compransoris* | | | | | | *Zavarzinia compransoris* (JX986958) | | | | | | 94.65 | | | Proteobacteria | | | Acetobacteraceae | | | |  |  |  |
|  |  | EGA240375 | | | *Bacillus subtilis* | | | | | | *Bacillus tequilensis* (AYTO01000043) | | | | | | 99.93 | | | Firmicutes | | | Bacillaceae | | | |  |  |  |
|  |  | EGA240376 | | | *Bacillus subtilis* | | | | | | *Bacillus tequilensis* (AYTO01000043) | | | | | | 99.72 | | | Firmicutes | | | Bacillaceae | | | |  |  |  |
|  |  | EGA240377 | | | *Stenotrophomonas* sp. | | | | | | CP026001_s (CP026001) | | | | | | 99.44 | | | Proteobacteria | | | Xanthomonadaceae | | | |  |  |  |
| root | | EGA240378 | | | *Phycicoccus jejuensis* | | | | | | *Phycicoccus jejuensis* (JOEE01000020) | | | | | | 99.72 | | | Actinobacteria | | | Lntrasporangiaceae | | | |  |  |  |
|  |  | EGA240379 | | | *Bacillus subtilis* | | | | | | *Bacillus tequilensis* (AYTO01000043) | | | | | | 99.79 | | | Firmicutes | | | Bacillaceae | | | |  |  |  |
|  |  | EGA240380 | | | *Bacillus subtilis* | | | | | | *Bacillus tequilensis* (AYTO01000043) | | | | | | 99.58 | | | Firmicutes | | | Bacillaceae | | | |  |  |  |
|  |  | EGA240381 | | | *Bacillus subtilis* | | | | | | *Bacillus tequilensis* (AYTO01000043) | | | | | | 99.65 | | | Firmicutes | | | Bacillaceae | | | |  |  |  |
|  |  | EGA240382 | | | *Rhizobium sp.* strain MERSZ-6 | | | | | | *Rhizobium wuzhouense* (MG857114) | | | | | | 99.78 | | | Proteobacteria | | | Rhizobiaceae | | | |  |  |  |
|  |  | EGA240383 | | | *Bacillus* sp. S2(2014) | | | | | | *Bacillus tequilensis* (AYTO01000043) | | | | | | 99.72 | | | Firmicutes | | | Bacillaceae | | | |  |  |  |
|  |  | EGA240384 | | | *Bacillus subtilis* | | | | | | *Bacillus tequilensis* (AYTO01000043) | | | | | | 99.58 | | | Firmicutes | | | Bacillaceae | | | |  |  |  |
|  |  | EGA240386 | | | *Bacillus subtilis* | | | | | | *Bacillus tequilensis* (AYTO01000043) | | | | | | 99.70 | | | Firmicutes | | | Bacillaceae | | | |  |  |  |
|  |  | EGA240387 | | | *Bacillus subtilis* | | | | | | *Bacillus tequilensis* (AYTO01000043) | | | | | | 99.86 | | | Firmicutes | | | Bacillaceae | | | |  |  |  |
|  |  | EGA240388 | | | *Bacillus subtilis* | | | | | | *Bacillus tequilensis* (AYTO01000043) | | | | | | 99.51 | | | Firmicutes | | | Bacillaceae | | | |  |  |  |
|  |  | EGA240389 | | | *Bacillus subtilis* | | | | | | *Bacillus tequilensis* (AYTO01000043) | | | | | | 99.51 | | | Firmicutes | | | Bacillaceae | | | |  |  |  |
|  |  | EGA240390 | | | *Bacillus amyloliquefaciens* | | | | | | *Bacillus tequilensis* (AYTO01000043) | | | | | | 99.93 | | | Firmicutes | | | Bacillaceae | | | |  |  |  |
|  |  | EGA240391 | | | *Microbacterium* sp. HBUM179310 | | | | | | *Microbacterium hydrothermale* (HM222660) | | | | | | 98.63 | | | Actinobacteria | | | Microbacteriaceae | | | |  |  |  |
|  |  | EGA240393 | | | *Bacillus subtilis* | | | | | | *Bacillus tequilensis* (AYTO01000043) | | | | | | 99.72 | | | Firmicutes | | | Bacillaceae | | | |  |  |  |
|  |  | EGA240394 | | | *Rhizobium* sp. HBU08115 | | | | | | *Rhizobium oryzicola* ([JX446583](https://www.ezbiocloud.net/16SrRNA?ac=JX446583)) | | | | | | 99.45 | | | Proteobacteria | | | Rhizobiaceae | | | |  |  |  |
|  |  | EGA240395 | | | *Mycobacterium* sp. M26 | | | | | | LN929908_s (LN929908) | | | | | | 99.36 | | | Actinobacteria | | | Mycobacteriaceae | | | |  |  |  |
|  |  | EGA240396 | | | *Rhizobium* sp. | | | | | | *Rhizobium aquaticum* (KM083136) | | | | | | 99.61 | | | Proteobacteria | | | Rhizobiaceae | | | |  |  |  |
|  |  | EGA240397 | | | *Bacillus subtilis* | | | | | | *Bacillus tequilensis* (AYTO01000043) | | | | | | 99.44 | | | Firmicutes | | | Bacillaceae | | | |  |  |  |
|  |  | EGA240398 | | | *Bacillus velezensis* | | | | | | *Bacillus siamensis* ([AJVF01000043](https://www.ezbiocloud.net/16SrRNA?ac=AJVF01000043)) | | | | | | 99.86 | | | Firmicutes | | | Bacillaceae | | | |  |  |  |
|  |  | EGA240399 | | | *Bacillus subtilis* | | | | | | *Bacillus tequilensis* (AYTO01000043) | | | | | | 99.93 | | | Firmicutes | | | Bacillaceae | | | |  |  |  |
|  |  | EGA240400 | | | *Priestia megaterium* | | | | | | *Priestia aryabhattai* (EF114313) | | | | | | 99.86 | | | Firmicutes | | | Bacillaceae | | | |  |  |  |
|  |  | EGA240401 | | | *Chryseobacterium* sp. zd2 | | | | | | *Chryseobacterium massiliae* (AF531766) | | | | | | 99.40 | | | Bacteroidetes | | | Weeksellaceae | | | |  |  |  |
|  |  | EGA240402 | | | Uncultured *Xanthobacter* sp. | | | | | | *Aquabacter spiritensis* (FR733686) | | | | | | 98.25 | | | Proteobacteria | | | Xanthobacteraceae | | | |  |  |  |
|  |  | EGA240403 | | | *Aeromonas veronii* | | | | | | *Aeromonas veronii* (CDDK01000015) | | | | | | 99.02 | | | Proteobacteria | | | Aeromonadaceae | | | |  |  |  |
|  |  | EGA240404 | | | *Pseudomonas oryzihabitans* | | | | | | *Pseudomonas oryzihabitans* (BBIT01000012) | | | | | | 99.02 | | | Proteobacteria | | | Pseudomonadaceae | | | |  |  |  |
|  |  | EGA240405 | | | *Bacillus subtilis* | | | | | | *Bacillus tequilensis* (AYTO01000043) | | | | | | 99.37 | | | Firmicutes | | | Bacillaceae | | | |  |  |  |
|  |  | EGA240406 | | | *Fictibacillus* sp. | | | | | | Fictibacillus halophilus (KP265300) | | | | | | 99.93 | | | Firmicutes | | | Bacillaceae | | | |  |  |  |
|  |  | EGA240407 | | | *Bacillus* sp. (in: Bacteria) | | | | | | *Bacillus tequilensis* (AYTO01000043) | | | | | | 99.65 | | | Firmicutes | | | Bacillaceae | | | |  |  |  |
|  |  | EGA240408 | | | *Roseomonas* sp. | | | | | | *Roseomonas lacus* (AJ786000) | | | | | | 96.27 | | | Proteobacteria | | | Acetobacteraceae | | | |  |  |  |
|  |  | EGA240409 | | | *Bacillus subtilis* | | | | | | *Bacillus tequilensis* (AYTO01000043) | | | | | | 99.51 | | | Firmicutes | | | Bacillaceae | | | |  |  |  |
|  |  | EGA240410 | | | *Bacillus* sp MN13 | | | | | | *Bacillus tequilensis* (AYTO01000043) | | | | | | 99.37 | | | Firmicutes | | | Bacillaceae | | | |  |  |  |

| **Table S3** | Microbiological validation of OTUs of endophytic bacteriome in *E. crassipes*. |
| --- | --- |

| **Genus** | **Bacteriome based on 16S rRNA gene sequencing** | | | | | **Microbiological tools** | | | | |
| --- | --- | --- | --- | --- | --- | --- | --- | --- | --- | --- |
|  | Root | Stem | Leaf | Total OTUs | Frequency (%) | Root | Stem | Leaf | Total isolates (n) | Frequency (%) |
| *Bacillus* | 3598 | 82 | 568 | 4248 | 100 | 18 | 6 | 6 | 30 | 100 |
| *Cytophaga* | – | – | – | – | – | – | – | 1 | 1 | 33.3 |
| *Roseateles* | – | – | – | – | – | – | – | 1 | 1 | 33.3 |
| *Herbaspirillum* | 58 | 5932 | 8872 | 14862 | 100 | – | – | 1 | 1 | 33.3 |
| *Acinetobacter* | 0 | 1 | 5 | 6 | 66.7 | – | – | 3 | 3 | 33.3 |
| *Agrobacterium* | – | – | – | – | – | – | – | 1 | 1 | 33.3 |
| *Sphingomonas* | 874 | 4716 | 15406 | 20996 | 100 | – | – | 2 | 2 | 33.3 |
| *Microbacterium* | 141 | 98 | 863 | 1102 | 100 | 1 | – | 1 | 2 | 66.7 |
| *Zavarzinia* | – | – | – | – | – | – | 1 | – | 1 | 33.3 |
| *Stenotrophomonas* | 86 | 112 | 284 | 482 | 100 | – | 1 | – | 1 | 33.3 |
| *Phycicoccus* | 10 | 15 | 135 | 160 | 100 | 1 | – | – | 1 | 33.3 |
| *Allorhizobium-Neorhizobium-Pararhizobium-Rhizobium* | 558 | 597 | 15891 | 17046 | 100 | 3 | – | – | 3 | 33.3 |
| *Mycobacterium* | 937 | 84 | 246 | 1267 | 100 | 1 | – | – | 1 | 33.3 |
| *Priestia* | – | – | – | – | – | 1 | – | – | 1 | 33.3 |
| *Chryseobacterium* | 7 | 195 | 1007 | 1209 | 100 | 1 | – | – | 1 | 33.3 |
| *Aquabacter* | – | – | – | – | – | 1 | – | – | 1 | 33.3 |
| *Aeromonas* | 123 | 32 | 90 | 245 | 100 | 1 | – | – | 1 | 33.3 |
| *Pseudomonas* | 141 | 336 | 1981 | 2458 | 100 | 1 | – | – | 1 | 33.3 |
| *Fictibacillus* | 45 | 0 | 1 | 46 | 66.7 | 1 | – | – | 1 | 33.3 |
| *Roseomonas* | 1357 | 50 | 119 | 1526 | 100 | 1 | – | – | 1 | 33.3 |

| **Table S4** | *In vitro* functional profile of bacterial isolates from *E. crassipes* on plant growth promotions traits. |
| --- | --- |

| **Isolate name** | **Genus** | **ACC deaminase** | **Organic phosphorus solubilization** | **Inorganic phosphorus solubilization** | **Siderophore** | **IAA** | **Nitrogen fixation** | **Cellulase** | **Chitinase** | **Protease** |
| --- | --- | --- | --- | --- | --- | --- | --- | --- | --- | --- |
| EGA240353 | *Bacillus* | + |  |  |  |  |  | + | + | + |
| EGA240354 | *Bacillus* | + |  |  |  | + |  | + |  |  |
| EGA240355 | *Bacillus* | + |  | + |  | + |  | + | + | + |
| EGA240356 | *Bacillus* | – |  | + |  | + | + |  |  | + |
| EGA240357 | *Cytophaga* | – | + | + | + | + |  |  | + |  |
| EGA240358 | *Roseateles* | + |  | + |  | + | + |  |  |  |
| EGA240359 | *Herbaspirillum* | – |  |  |  |  | + |  |  |  |
| EGA240360 | *Bacillus* | – |  | + |  |  |  | + |  | + |
| EGA240361 | *Acinetobacter* | – | + |  | + | + |  |  |  |  |
| EGA240363 | *Acinetobacter* | – | + | + | + | + |  |  |  |  |
| EGA240364 | *Acinetobacter* | + | + | + | + | + |  |  |  |  |
| EGA240365 | *Agrobacterium* | – |  | + |  |  |  |  |  |  |
| EGA240366 | *Sphingomonas* | – | + | + |  |  |  |  |  |  |
| EGA240367 | *Bacillus* | + |  |  |  | + |  |  |  |  |
| EGA240368 | *Sphingomonas* | + | + | + |  |  |  |  |  |  |
| EGA240369 | *Microbacterium* | – | + | + |  |  |  |  |  |  |
| EGA240370 | *Bacillus* | + |  | + |  | + |  | + |  | + |
| EGA240371 | *Bacillus* | + |  | + |  |  |  | + | + |  |
| EGA240372 | *Bacillus* | + |  |  |  | + |  | + |  | + |
| EGA240373 | *Bacillus* | – |  |  |  | + |  |  |  |  |
| EGA240374 | *Zavarzinia* | + | + | + |  |  |  | + |  |  |
| EGA240375 | *Bacillus* | – |  |  |  | + |  |  | + | + |
| EGA240376 | *Bacillus* | – |  | + |  | + |  | + |  |  |
| EGA240377 | *Stenotrophomonas* | – |  |  | + | + |  |  |  | + |
| EGA240378 | *Phycicoccus* | – |  | + | + | + |  |  |  |  |
| EGA240379 | *Bacillus* |  |  | + |  | + |  | + | + | + |
| EGA240380 | *Bacillus* |  |  | + | + | + |  |  | + | + |
| EGA240381 | *Bacillus* | + |  |  |  |  |  | + |  |  |
| EGA240382 | *Rhizobium* | + |  | + |  |  | + |  |  |  |
| EGA240383 | *Bacillus* |  |  |  |  | + |  | + |  | + |
| EGA240384 | *Bacillus* | + |  |  |  | + | + |  | + | + |
| EGA240386 | *Bacillus* |  |  | + |  | + |  | + |  | + |
| EGA240387 | *Bacillus* |  |  |  |  | + |  | + | + |  |
| EGA240388 | *Bacillus* | + |  | + | + |  |  | + |  | + |
| EGA240389 | *Bacillus* |  |  |  |  | + |  |  | + |  |
| EGA240390 | *Bacillus* | + |  | + |  | + |  |  | + |  |
| EGA240391 | *Microbacterium* | + | + | + | + |  |  |  |  |  |
| EGA240393 | *Bacillus* |  |  |  |  |  |  | + | + | + |
| EGA240394 | *Rhizobium* | + |  | + |  |  | + |  |  |  |
| EGA240395 | *Mycobacterium* |  | + |  | + |  |  |  |  |  |
| EGA240396 | *Rhizobium* | + |  | + |  | + | + |  |  |  |
| EGA240397 | *Bacillus* |  |  |  |  |  |  | + | + | + |
| EGA240398 | *Bacillus* | + |  | + |  | + | + | + | + | + |
| EGA240399 | *Bacillus* |  |  |  | + | + |  | + | + |  |
| EGA240400 | *Priestia* | + | + | + |  |  |  |  |  |  |
| EGA240401 | *Chryseobacterium* | + | + |  |  |  |  |  |  |  |
| EGA240402 | *Aquabacte* | + | + | + |  |  | + |  | + |  |
| EGA240403 | *Aeromonas* | + | + |  |  |  |  |  |  |  |
| EGA240404 | *Pseudomonas* | + | + | + | + | + | + | + |  | + |
| EGA240405 | *Bacillus* |  |  | + |  |  |  | + |  | + |
| EGA240406 | *Fictibacillus* | + |  |  |  | + | + |  | + |  |
| EGA240407 | *Bacillus* |  |  | + |  |  |  | + | + |  |
| EGA240408 | *Roseomonas* | + | + | + | + | + |  |  |  |  |
| EGA240409 | *Bacillus* |  |  |  |  | + |  |  | + | + |
| EGA240410 | *Bacillus* |  |  |  |  | + |  | + | + | + |
